# Rapid deep ocean deoxygenation and acidification threaten life on Northeast Pacific seamounts

**DOI:** 10.1111/gcb.15307

**Published:** 2020-09-14

**Authors:** Tetjana Ross, Cherisse Du Preez, Debby Ianson

**Affiliations:** ^1^ Institute of Ocean Sciences, Fisheries and Oceans Canada (DFO) Sidney BC Canada

**Keywords:** benthic ecosystems, climate change, cold water corals, ecosystem‐based management, ocean acidification, ocean biogeochemistry, ocean deoxygenation, vulnerable marine ecosystems

## Abstract

Anthropogenic climate change is causing our oceans to lose oxygen and become more acidic at an unprecedented rate, threatening marine ecosystems and their associated animals. In deep‐sea environments, where conditions have typically changed over geological timescales, the associated animals, adapted to these stable conditions, are expected to be highly vulnerable to any change or direct human impact. Our study coalesces one of the longest deep‐sea observational oceanographic time series, reaching back to the 1960s, with a modern visual survey that characterizes almost two vertical kilometers of benthic seamount ecosystems. Based on our new and rigorous analysis of the Line P oceanographic monitoring data, the upper 3,000 m of the Northeast Pacific (NEP) has lost 15% of its oxygen in the last 60 years. Over that time, the oxygen minimum zone (OMZ), ranging between approximately 480 and 1,700 m, has expanded at a rate of 3.0 ± 0.7 m/year (due to deepening at the bottom). Additionally, carbonate saturation horizons above the OMZ have been shoaling at a rate of 1–2 m/year since the 1980s. Based on our visual surveys of four NEP seamounts, these deep‐sea features support ecologically important taxa typified by long life spans, slow growth rates, and limited mobility, including habitat‐forming cold water corals and sponges, echinoderms, and fish. By examining the changing conditions within the narrow realized bathymetric niches for a subset of vulnerable populations, we resolve chemical trends that are rapid in comparison to the life span of the taxa and detrimental to their survival. If these trends continue as they have over the last three to six decades, they threaten to diminish regional seamount ecosystem diversity and cause local extinctions. This study highlights the importance of mitigating direct human impacts as species continue to suffer environmental changes beyond our immediate control.

## Graphical abstract

Climate change is influencing ocean oxygen and carbon concentrations in a manner and at a pace that will impact life on seamounts in the deep sea, where environmental conditions are usually remarkably constant, and animals often live for hundreds of years. The Northeast Pacific, home to both an oxygen minimum zone and rich seamount communities, is rapidly losing oxygen (15% since 1960), and carbonate saturation horizons are shoaling at 1–2 m/year. Changing conditions within the narrow depth ranges of long‐lived species threaten their survival. If trends continue, within a few decades local extinctions will become common as ecosystem diversity declines.

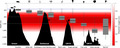

## 1 INTRODUCTION

Anthropogenic climate change is rapidly altering physical and chemical conditions in the ocean (Gruber, 2011); however, impacts on marine life, from which humankind derives significant cultural, social, and economic value (Costanza, 1999), remain largely uncertain (Haigh, Ianson, Holt, Neate, & Edwards, 2015; Hoegh‐Guldberg & Bruno, 2010). On a global scale, the ocean is warming (Meyssignac et al., 2019; Wijffels, Roemmich, Monselesan, Church, & Gilson, 2016), losing oxygen (Breitburg et al., 2018; Schmidtko, Stramma, & Visbeck, 2017), and absorbing carbon dioxide (making it more acidic; Gruber et al., 2019; Raven et al., 2005). Many species will relocate in response (Pinsky, Worm, Fogarty, Sarmiento, & Levin, 2013), but some species cannot. In particular, long‐lived, habitat‐forming deep‐sea species that are adapted to remarkably constant conditions, which typically change over geological timescales (Danovaro, Corinaldesi, Dell’Anno, & Snelgrove, 2017), may not be able to keep pace with these ocean changes.

Seamounts can be viewed as a microcosm for the impact of climate change on benthic ecosystems. They occur worldwide (Yesson et al., 2020), are often isolated, consist of relatively uniform volcanic substrates that, due to natural gradients in water properties, span a huge range of environmental conditions and habitats, and are home to many species that are fixed in place. Additionally, they are oases of life in the deep ocean, as they enhance primary productivity, concentrate local productivity, and provide refugia for some continental slope species (Rowden, Dower, Schlacher, Consalvey, & Clark, 2010). Thus, they are considered essential ecosystems in need of protection (IUCN, 2017). It has been long established that mountains are vulnerable to climate change (Huber, Bugmann, & Reasoner, 2006), changing faster than other terrestrial habitats (Nogués‐Bravo, Araújo, Errea, & Martinez‐Rica, 2007). Although understudied, as underwater mountains, the same might be expected for seamounts.

There is a large cluster of seamounts (undersea mountain >1,000 m tall) in the Northeast Pacific (NEP). These seamounts, with varying summit depths and geomorphologies, form one of the densest undersea mountain ranges in the world. This region is also home to a large oxygen minimum zone (OMZ), between ~480 and 1,700 m depth, which contains some of the lowest oxygen levels in the global ocean (Paulmier & Ruiz‐Pino, 2009). This water is “old,” that is, has long been away from the surface (~1,000 years, Gebbie & Huybers, 2012), and has experienced significant biological consumption (Talley, 2011). Many of the seamounts intersect the OMZ, so their summits and flanks pass through a wide range of environmental conditions. The remaining seamounts contend with the OMZ as a chemical ceiling. Benthic organisms are generally found in stratified bands encircling the seamounts (Du Preez, Curtis, & Clarke, 2016), the depths of which are determined by both environmental (e.g., temperature, oxygen; Wishner, Levin, Gowing, & Mullineaux, 1990) and biological (e.g., competition; Victorero, Robert, Robinson, Taylor, & Huvenne, 2018) factors.

In addition to the already low oxygen levels, this region has been experiencing loss of oxygen, that is, deoxygenation, faster than the global average (Cummins & Ross, 2020; Schmidtko et al., 2017). Whitney, Freeland, and Robert (2007) reported significant shoaling of the 1.5 m/L oxygen horizon in the NEP. Changes in oxygen levels are expected to alter seamount ecosystems and their associated communities (Clark, Watling, Rowden, Guinotte, & Smith, 2011; Levin, 2003; Wishner et al., 1990).

Concurrent with deoxygenation, total carbon dioxide (DIC) is increasing in the NEP, because carbon and oxygen are so closely linked through respiration (Anderson & Sarmiento, 1994) and due to uptake of anthropogenic carbon from the atmosphere (Gruber et al., 2019; Sabine et al., 2004). Increased carbon causes significant changes in ocean carbon chemistry, including increased acidity (reduction in pH; termed “ocean acidification,” OA, Caldeira & Wickett, 2003; IPCC, 2011), and may negatively impact marine organisms in multiple ways (Haigh et al., 2015). The calcium carbonate saturation state (Ω) provides a chemical measure of the energetic cost for calcification, specific to polymorph (Supporting Information, Section S1). It places a constraint on calcifying organisms (e.g., Andersson, Mackenzie, & Bates, 2008; Spalding, Finnegan, & Fischer, 2017) because they must manipulate local chemistry to build and maintain their structures (Adkins, Boyle, Curry, & Lutringer, 2003; Weiner & Dove, 2003). OA increases these energetic costs by reducing Ω (Spalding et al., 2017). The present‐day surface ocean is usually supersaturated (Ω > 1), becoming less saturated with depth, driven mainly by increases in pressure and DIC (Jiang et al., 2015; Li, Takahashi, & Broecker, 1969). Calcium carbonate polymorphs in direct contact with seawater below their associated saturation horizons (Ω = 1) will dissolve. Saturation horizons are already remarkably shallow in the old, carbon‐rich NEP relative to other ocean basins (Feely et al., 2004) and are getting shallower (Feely et al., 2012).

**FIGURE 1:**
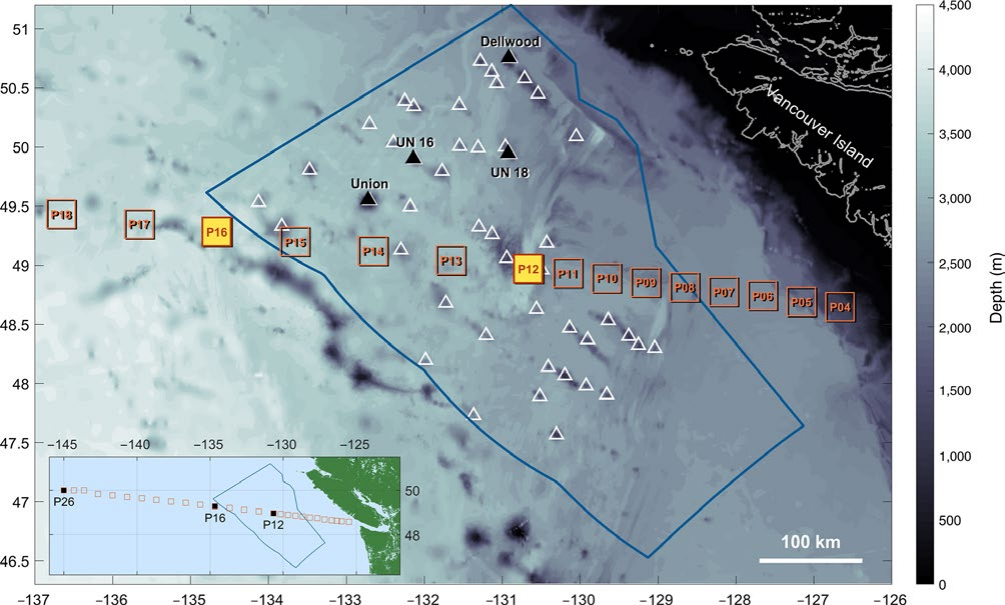
Map of our Northeast Pacific seamounts study region. Line P oceanographic monitoring stations are indicated by red squares; major stations (P12, P16, and P26) are highlighted in yellow (main map) and black (inset). The triangle symbols indicate seamount summits in our study region (DFO, 2019), the four in black were surveyed during the 2017 benthic imaging survey cruise (Table 1). The proposed Offshore Pacific marine protected area is outlined in blue. Bathymetry is from GEBCO 2014 Grid, version 20141103, http://www.gebco.net

Here, we focus on an NEP seamounts study region (Figure 1) that contains a dense cluster of 47 seamounts, 32 of which intersect the OMZ (oxygen < 1 ml/L), and is bisected by the long‐term oceanographic monitoring section, Line P. We calculate trends in oxygen from 1960 to present in the deep ocean bathing these seamounts and examine the mechanisms driving these trends. Similarly, we calculate deep Ω (aragonite and calcite) trends from 1987 to present. We explicitly resolve the evolution of the OMZ and saturation horizons in time, while critically investigating uncertainties in the data and derived trends. Modern benthic seamount survey data from four of the seamounts in the region were analyzed to identify key indicators, including cold water corals, sponges, echinoderms, and rockfish, based on their depth distributions relative to the chemical horizons. We identify potential implications of oxygen and Ω trends to these key species considering their life histories and mineral compositions. Finally, we discuss these trends in the context of ecosystem diversity and marine protected area (MPA) planning, which is timely as this NEP seamount cluster is a significant component of a proposed MPA (DFO, 2019).

## 2 METHODS

### 2.1 Oceanographic data

The oceanographic data were collected as part of the Line P monitoring program (Figure 1), starting in 1959 and sampled at least three times a year (Cummins & Masson, 2012; Freeland, 2007; Mackas & Galbraith, 2012; Whitney et al., 2007). Discrete bottle chemistry data were collected throughout the time series along with electronic sensor measurements (i.e., CTD observations) starting in 1969 for temperature and salinity and in 2001 for oxygen (Seabird 43 dissolved oxygen sensor). For all data (oceanographic and ecological), we refer to depth rather than pressure (i.e., 1 dbar = 1 m) for convenience, even though at 3,000 dbar, this simplified conversion is off by ~1.5%. Bottle samples have been analyzed for oxygen (present: automated Winkler Photometric System) and macronutrient (present: Technicon Autoanalyzer II) concentrations since 1959, and are collected from 24 discrete depths at each major station (Figure 1). Since 1987, a subset of the bottle samples have been analyzed for dissolved inorganic carbon (DIC) concentration and total alkalinity (TA) following the standard sampling and analysis protocol (Dickson & Goyet, 1994; Dickson, Sabine, & Christian, 2007; S1). TA becomes more accurate later in the record when the open‐cell alkalinity system (Dickson et al., 2007) was adopted. Like salinity, dilution (precipitation and evaporation) exerts the primary control over variations in ocean TA (Millero, Lee, & Roche, 1998), allowing us to use the recent TA observations to extend the record back in time to cover the full DIC time series using empirical relationships (S2).

We use Ω to indicate the biological impact of increasing DIC because Ω directly impacts calcifying marine organisms like corals (Kleypas et al., 1999) and echinoderms (Dupont, Ortega‐Martinez, & Thorndyke, 2010) and because Ω and acidity (pH—which also affects animal physiology; Claiborne, Edwards, & Morrison‐Shetlar, 2002) covary at the depths and salinity ranges in our study zone. Saturation states of two common biogenic calcium carbonate mineral forms, aragonite (Ω_Ar_) and calcite (Ω_Ca_), were calculated using measured DIC, empirically derived TA and coincident observations of temperature, salinity, phosphate and silicate using CO2SYS (Orr, Epitalon, Dickson, & Gattuso, 2018; Van Heuven, Pierrot, Rae, Lewis, & Wallace, 2011; S1). High‐magnesium‐calcite, a common, but heterogeneous, biogenic form is even more soluble than aragonite and calcite, but its exact solubility dependence is not certain (Andersson et al., 2008; Davis, Dove, & De Yoreo, 2000).

#### 2.1.1 Data analysis

Monthly, 5 m gridded temperature, salinity, density, oxygen and DIC data were prepared for each station (Figure 1) following Cummins and Ross (2020). Given the longer time series and more regular sampling of oxygen, the monthly oxygen data were averaged by year for subsequent analysis. The uncertainties calculated for the time series in each depth bin at each station reflected mainly the standard deviation between profiles for oxygen (intra‐annual variability) and the uncertainties in the observations and constants used to determine the carbon system for Ω (S3).

Next, the data for the stations were averaged spatially to represent the changes over our NEP seamounts study region. Across all stations, oxygen below about 300 m behaved similarly with depth; thus, we use an uncertainty‐weighted average across the stations P06–P16 (within the seamount study region) but also included station P26 to take advantage of its much more continuous time series, thereby creating a single profile which represents the entire region for each year from 1959 to present. A caveat, in some early years, the oxygen is represented by only the distant station P26. For DIC, there are longitudinal trends in the full data (S2), so we only use data from within the study region (P12 and P16, Figure 1) to compute the area‐average Ω profiles. The uncertainties in oxygen and Ω were propagated and combined with the spatial standard deviations (S3).

Multiple definitions of “hypoxic” appear in the literature (from 0.5 to 1.5 ml/L; e.g., Breitburg et al., 2018; Levin, 2003; Paulmier & Ruiz‐Pino, 2009; Whitney et al., 2007). We define the OMZ as water with less than 1 ml/L oxygen (Clark et al., 2011) and label these waters as hypoxic and those with less than 0.5 ml/L as severely hypoxic. Depths of the top and bottom of the OMZ and carbonate saturation state horizons (Ω_Ca_ = 1 and Ω_Ar_ = 1, 0.7) were determined for each oxygen/Ω profile and averaged over time and then space just like the study region averaged profiles.

Finally, all trends were calculated using a weighted least squares linear fit and the uncertainty on the trend estimated using bootstrapping (Efron & Gong, 1983). Trends in oxygen at fixed density were calculated by creating a regular density grid and using the coincident density profile to map oxygen onto that grid at each time step, then extracting data from a constant density bin. To average over the depth distributions of the indicator taxa (see Section 2.2 for definition), the depth distribution of the oxygen or Ω was averaged at each time step, weighting by the abundance of the animals in each depth bin. For all of these trends, the trend uncertainty was taken to be the 68% confidence intervals (equivalent to a standard deviation to combine it with uncertainty propagated from the temporal and spatial averages) as determined by bootstrapping; that is, randomly resampling (with replacement) the data to make 1,000 estimates of the weighted least‐squares linear fit and looking at the 16th and 84th percentiles.

### 2.2 Ecological data

Benthic ecology data were collected on the 2017 DFO expedition to Union, Dellwood, Unnamed (UN) 16, and UN 18 seamounts (Figure 1; Table 1) aboard the CCGS John P. Tully. Visual surveys of the benthic ecosystems were conducted from 1 m above the seafloor using a DFO drop camera system. BOOTS (Bathyal Ocean Observation and Televideo System) has a forward‐facing HD Minizeus video camera with 10 cm scaling lasers (Gale et al., 2017). Potential transects were randomly generated for each seamount “side,” with the final transect selection dependent on ocean and weather conditions (Table 1). A dive started with BOOTS at ~2,150 m (maximum equipment depth) in the morning, traveling slowly upslope (0.3–0.5 knots) to reach the summit by day's end (5–11 hr dives).

**TABLE 1:** Summary of 2017 seamount benthic imaging expedition (DFO‐Pac2017‐036)

	Union	Dellwood	UN 16	UN 18
Summit depth (m)	271	535	1,097	1,550
Latitude (summit)	49.546481	50.74695	49.88355	49.939332
Longitude (summit)	−132.702419	−130.89612	−132.113631	−130.905236
Distance to continental slope (km)	243	44	178	114
Dive survey				
Date(s)	21–24 July 2017	26–29 July 2017	25–26 July 2017	29 July 2017
No. of dives	5 (B022−26)	3 (B028−30)	1 (B027)	1 (B031)
Depths surveyed (m)	2,118–300	2,111–548	2,054–1,106	2,145–1,615
Distance surveyed (m)	21,400	19,100	27,00	3,000
Sides surveyed	SE, NE, & NW slopes & summit	N, E, SW slopes & summit	W slope & summit	NE slope & summit

We present depth (bathymetric) distributions of a subset of indicator taxa, derived from video annotation of abundance for all observed taxa as a function of depth. From the 10 BOOTS dives, 75.5 hr of video was annotated (protocol in Curtis et al., 2015), resulting in 265,135 1 s records, from which individual living organisms were resolved to the lowest taxonomic level possible with confidence, then georeferenced. In total, 27 taxonomic groupings of cold water corals and sponges were resolved, with an additional 78 taxa of other invertebrates and fish, between approximately 300 and 2,150 m depth. These data were merged into a single dataset, which was deemed suitable because taxa showed similar depth distribution independent of dive (S5). For each of these 105 taxa, the depth distribution was quantified by calculating the mean (*µ*), standard deviation (*σ*; a proxy for depth range size), and upper and lower limits.

#### 2.2.1 Indicator taxa

The large number of observed taxa was parsed down into nine indicators by focusing on taxonomic groups with narrow depth distributions in three key depth zones (defined below). The idea is that a narrow depth range on the steep seamount flanks represents a narrow ecological niche, since many marine environmental variables are correlated with depth. Specializing within a narrow depth range increases vulnerability to environmental change, potentially leading to extinction or extirpation (Gallagher, Hammerschlag, Cooke, Costa, & Irschick, 2015). In addition, the smaller the inhabitable isolated area, the less likely an immigration or recolonization rescue (MacArthur & Wilson, 2001). In comparison, a generalist species that can thrive within a large depth range is likely more versatile and resilient to change.

Because corals and sponges are often large, conspicuous, sessile animals, they are candidates as proxy indicators for ocean health and monitoring environmental changes (Girard & Fisher, 2018). Additionally, some of the same criteria that qualify them as defining features of Vulnerable Marine Ecosystems make them important in this study: they create unique/rare habitats; are of special importance for many species; are vulnerable and slow to recover; are biologically productive and diverse; and are structurally complex (CBD Decision IX/20 Annex^1^, ^2008^; FAO, 2009). Mobile species, especially those with high habitat fidelity, can have similar qualities (NAFO, 2018; O, Hannah, Greig, Boutillier, & Patton, 2015).

Three depth zones that have high ecological significance to seamounts within this region: (a) the surface to 800 m, the limit of direct nutrient flux from the surface at high latitudes (Knutsen, Wiebe, Gjøsæter, Ingvaldsen, & Lien, 2017), and a defining depth for classifying seamounts (Clark et al., 2011); (b) 800–1200 m, the region within the OMZ with severe hypoxia; that is, oxygen <0.5 ml/L (Gasbarro, Chu, & Tunnicliffe, 2019); and (c) deeper than 1,200 m, where dissolved oxygen increases.

The 105 taxa were parsed down to nine indicators by (a) retaining only the two mobile taxa with the narrowest depth distributions; (b) the two habitat‐forming cold water corals or sponges with the narrowest depth distributions in each of the depth zones: <800 m, 800–1200 m, and >1,200 m depth (where the average falls within the depth range); and finally, (c) a ubiquitous taxon for comparison.

## 3 RESULTS

### 3.1 Trends in oxygen, carbonate saturation state, and boundaries

**FIGURE 2:**
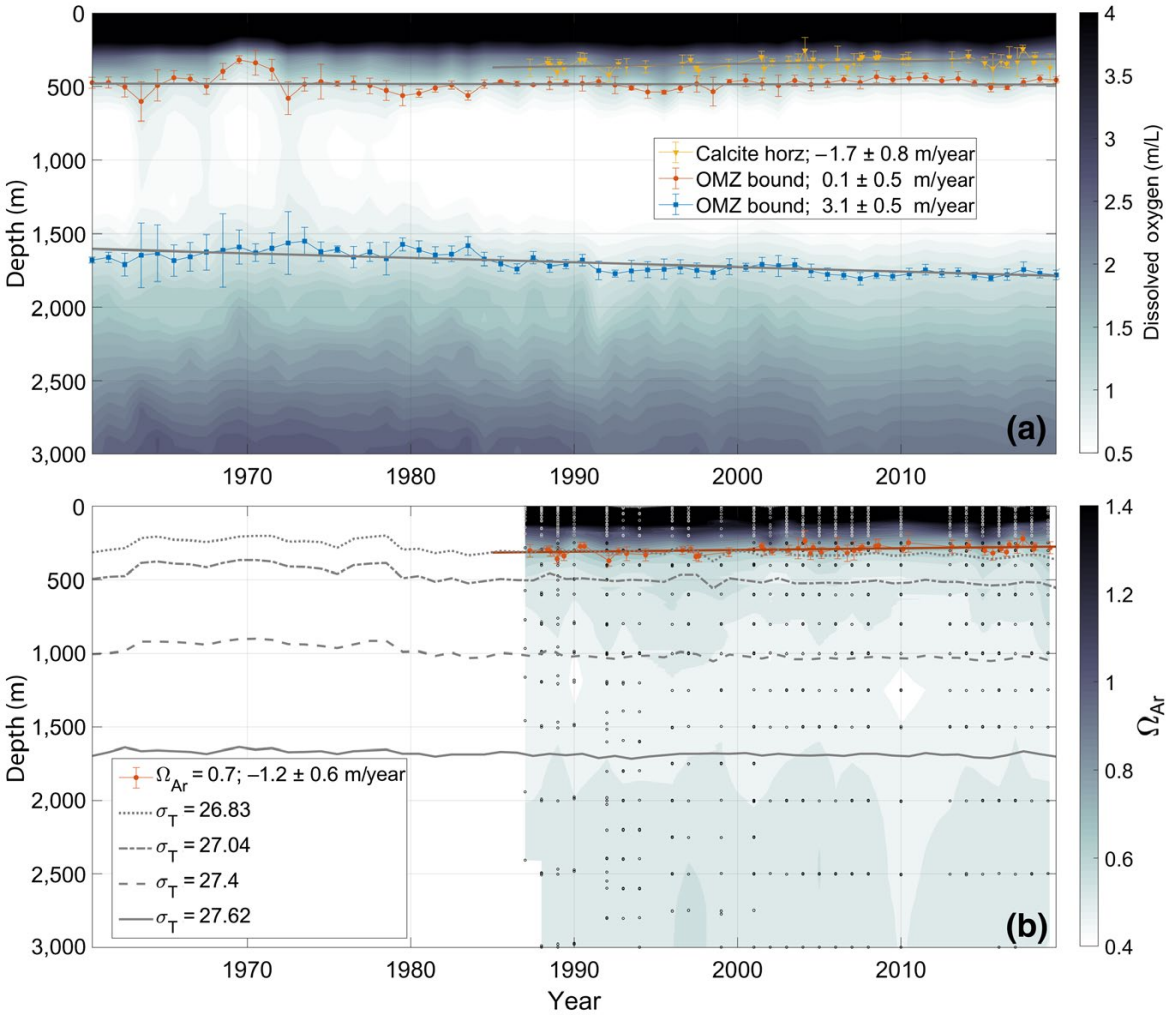
False color plots of dissolved oxygen (a) and aragonite saturation state (Ω_Ar_, b) over the Line P time series, averaged over our NEP seamounts study region (Figure 1). Overlaid on (a) are the calcite saturation depth (yellow), and the upper (red) and lower (blue) boundaries of the oxygen minimum zone. The legend indicates the trends associated with each of these boundaries. In (b), the Ω_Ar_ = 0.7 (red) horizon is shown. Also overlaid are the isopycnal depths for the 26.83 (thick gray dotted line), 27.04 (thick gray dash‐dotted line), 27.4 (thick gray dashed line), and 27.62 kg/m^3^ (thick gray line) sigma levels. Depths of the bottle samples that were interpolated to create the contour plot of Ω_Ar_ are shown (black dots)

The ocean bathing the NEP seamounts has been losing oxygen rapidly over the past 60 years, reducing the oxygen inventory in the upper 3,000 m by 15% (Figure 2). While showing considerable interannual variability, the upper bound of the OMZ has remained constant over the 60 year time series (mean ± standard deviation: 480 ± 50 m). In contrast, the bottom of the OMZ (mean: 1,700 m) has much smaller variability and shows a significant deepening trend (3.1 ± 0.5 m/year, Figure 2).

The seamounts are almost entirely surrounded by water undersaturated with respect to even the least soluble carbonate polymorph, calcite. The calcite saturation horizon (mean: 340 m) has shoaled at a rate of −1.7 ± 0.8 m/year since the late 1980s when carbon sampling began (Figure 2a). The aragonite saturation horizon is too shallow to intersect any of the seamounts in our study region (mean: 185 m), and is shoaling (1.0 ± 0.6 m/year, S4). The Ω_Ar_ = 0.7 horizon (mean: 300 m), a lower bound for corals elsewhere (e.g., Baco et al., 2017; Bostock et al., 2015), intersects some seamounts and is also shoaling (−1.2 ± 0.6 m/year; Figure 2). DIC decreases where oxygen increases below the OMZ, creating an “Ω minimum zone” between 1,000 and 1,500 m (Figure 2b), slightly deeper than the OMZ due to the strong pressure effects on Ω.

**FIGURE 3:**
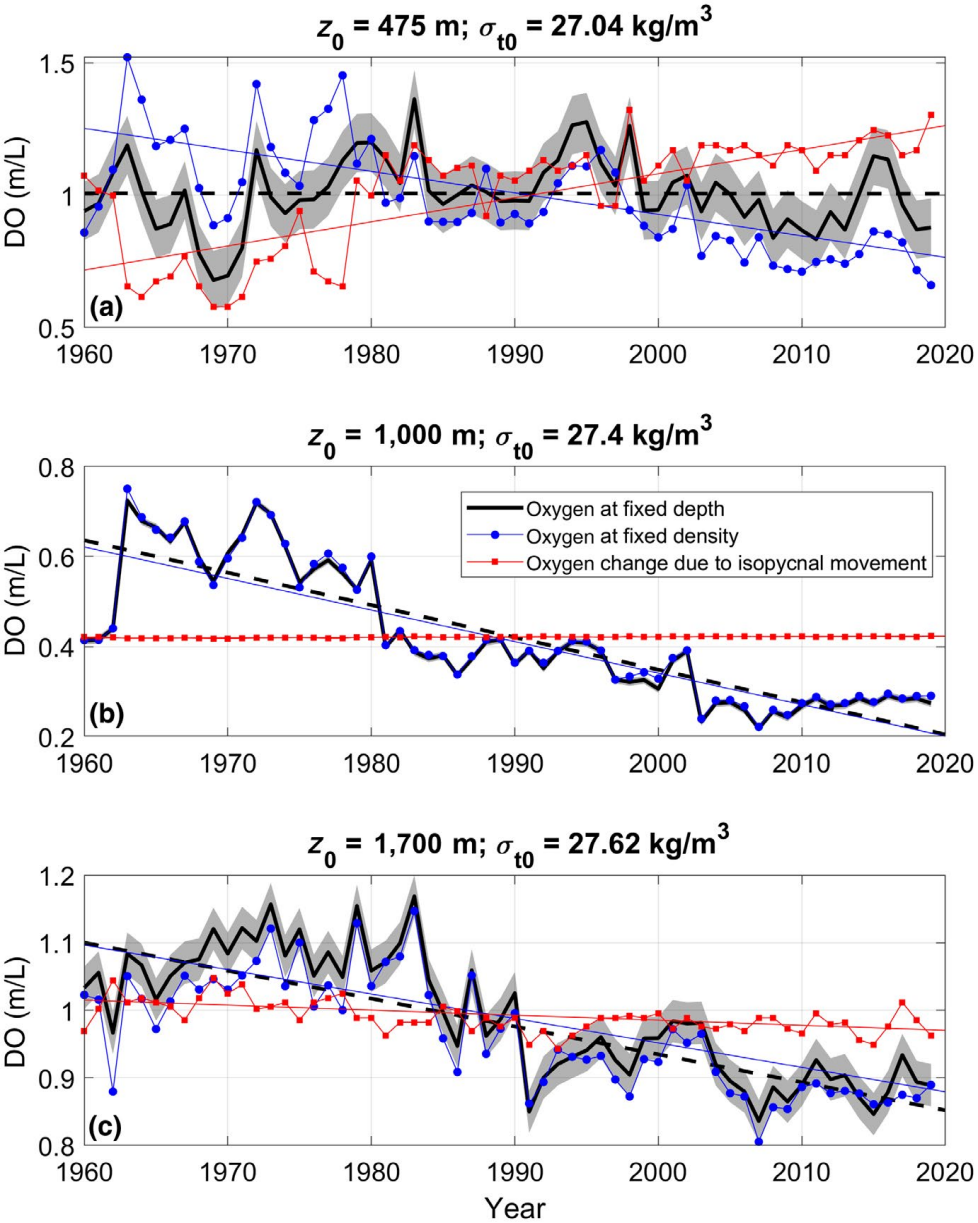
Time series of dissolved oxygen at fixed depth (475 m, a; 1,000 m, b; 1,700 m, c; thick black lines), at fixed potential density (1,027.04 kg/m^3^, a; 1,027.4 kg/m^3^, b; 1,027.62 kg/m^3^, c; blue lines/dots), and the estimated oxygen change due to movement of these constant potential density surfaces (mean oxygen has been added to put it on the same axis; red lines/squares). The gray patch behind the black lines represents the intra‐annual natural variability in the data at that depth (it is the inter‐month standard deviation from station P12 in 2013 [*N* = 4])

Basin‐scale changes in ocean circulation modulate oxygen loss and DIC gain at fixed depths. While oxygen is decreasing on isopycnals at all depths (blue circles, Figure 3), the downward movement of surfaces of constant water density (isopycnals), seen as deep as the middle of the OMZ (1,000 m; Figure 2b), can oppose that trend (red squares, Figure 3; following Cummins & Masson, 2012). For instance, the upper bound of the OMZ (~475 m) stayed fixed because the decreasing trend in oxygen on the isopycnal at that depth was balanced by an equal and opposite trend from the deepening isopycnals, bringing more oxygen to that depth (Figure 3a). The oxygen decline in the center of the OMZ (~1,000 m) was due to changes on an isopycnal (expected due to the negligible oxygen gradient in the center of the OMZ, so even if isopycnals are migrating, they bring no oxygen signal; Figure 3b). The oxygen trend at the bottom of the OMZ (~1,700 m) is primarily due to the trend on the mean isopycnal, not isopycnal motion (Figure 3c).

Similarly, Ω horizons between the top of the OMZ and the permanent pycnocline, where significant DIC accumulation is expected (Carter et al., 2019), are shoaling rapidly (Figure 2). Both Ω_Ar_ = 0.7 and Ω_Ca_ = 1 horizons exhibit strong trends despite the rapid deepening of isopycnals in that depth range (~2 m/year for 300–350 m; Figure 2b). Like the upper boundary of the OMZ, the iso‐Ω horizons would be shoaling even more quickly if the isopycnals were not deepening in this part of the water column. If the calcite saturation horizon continues to shoal at −1.7 m/year, all seamounts in this region will be bathed in water undersaturated with respect to all carbonate polymorphs by 2040. Below about 500 m, trends in Ω are hard to distinguish given the variability in the shorter time series (Figure 2).

### 3.2 Seamount indicator taxa, their depth ranges, and the chemical changes therein

**FIGURE 4:**
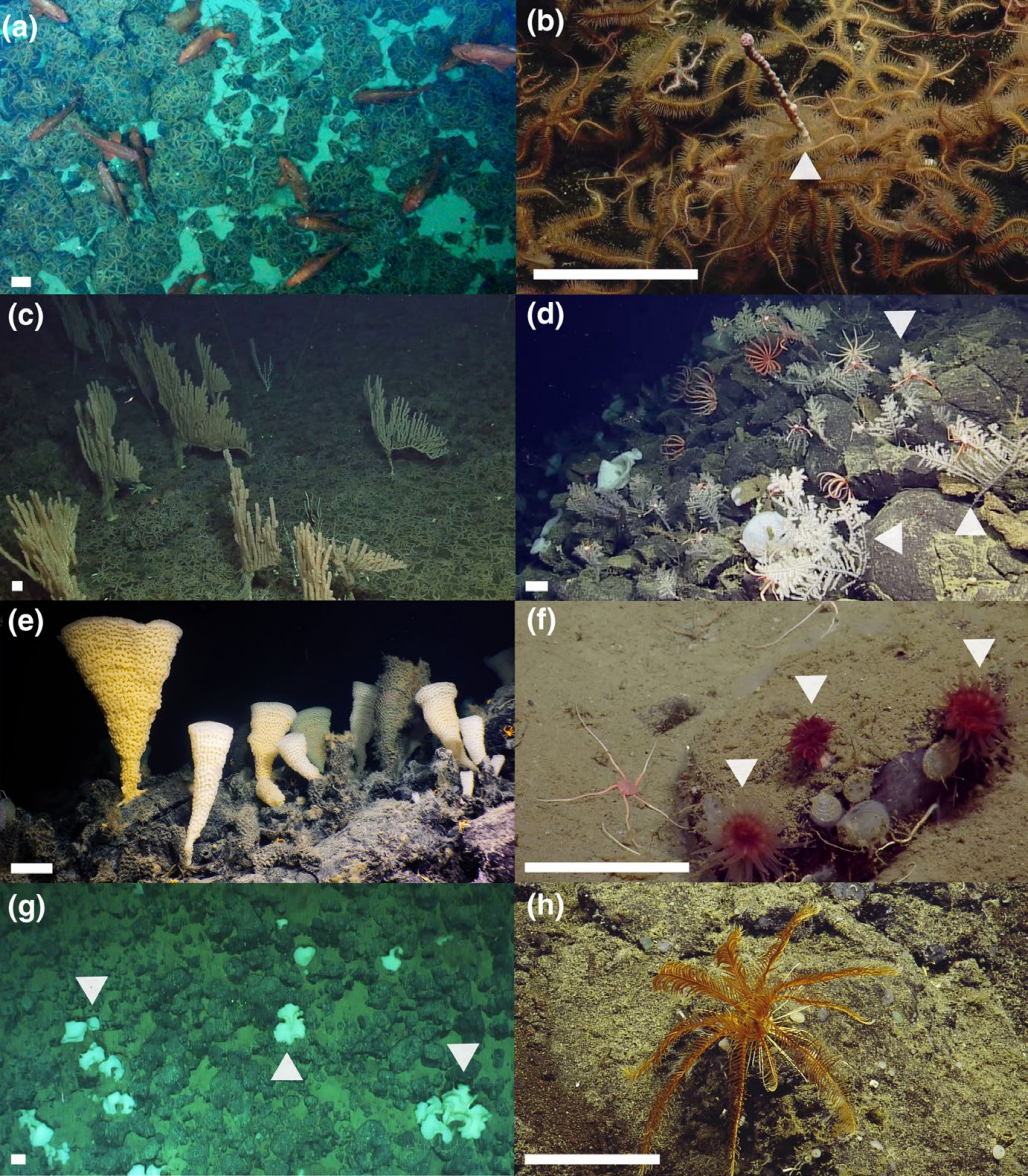
Images of the indicator taxa: (a) rougheye rockfish (*Sebastes aleutianus*) were observed as solitary individuals and in schools; (a–c) brittle stars (*Ophiacantha diplasia* and *Ophiopholis* spp.) could form dense living mats covering the seafloor often collocated with (also b, indicated with arrow) the bubblegum coral (*Paragorgia* cf. *jamesi*), which was small but dense while (c) the bamboo coral (*Isidella tentaculum*) was slightly less dense but could grow over 2 m high; (d, indicated with arrows) the black coral (*Chrysopathes speciosa*) formed extensive gardens; (e) the tall vase‐shape bugle sponge (*Pinulasma* n. sp.); (f, indicated with arrows) the cup coral (Flabellidae) is solitary coral; (g, indicated with arrows) the undulated glass sponges (cf *Tretodictyum* n. sp.) increased seafloor structural complexity with its body morphology; and (h) the sea lily was observed as solitary individuals and as dense fields (*Florometra serratissima*). The white scale bars in the corner of each panel are 10 cm long

**FIGURE 5:**
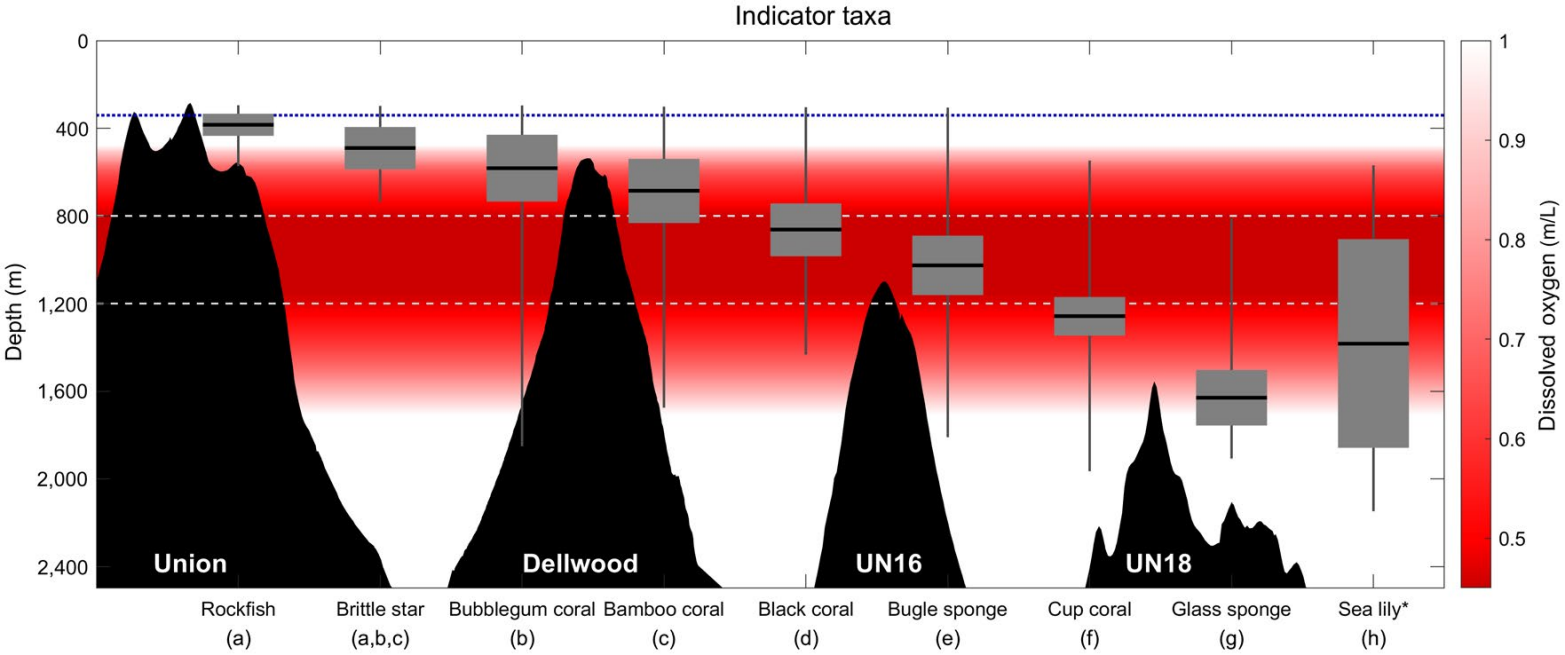
Plot of the observed depth distributions of the nine indicator taxa identified based on the benthic visual surveys along with simultaneously collected depth profiles of Union, Dellwood, Unnamed (UN) 16 and 18 seamounts (Figure 1). The red background shows the mean oxygen concentration over the full time series, highlighting the OMZ (i.e., white is outside the OMZ). The dashed white lines delineate the region between 800 and 1,200 m which corresponds to oxygen <0.5 ml/L and dotted blue line indicates the mean depth of the calcite saturation horizon. A range of metrics on the species’ depth ranges are plotted (mean, horizontal black lines; ±1 standard deviation, dark gray boxes; maximum and minimum depth observed, thin dark gray lines). *The sea lily has a bimodal depth distribution (gap between 1,150 and 700 m); the black coral also has a bimodal distribution (dip between 900 and 850 m); the rest have roughly bell‐shaped unimodal distributions (except for the rockfish, which is truncated at the top) (S5). All taxa were found on all seamounts with habitat (seafloor) in their depth range (mean ± 1 standard deviation) with the exception of the bugle sponge which was not observed on UN 16 (S5)

The exposed rocky flanks of the seamounts support dense forests of sessile, habitat‐forming, filter‐feeders such as soft corals (Alcyonacea), black corals (Antipatharia), hard corals (Scleractinian and hydrocorals), and sponges (Porifera), as well as abundant deep‐sea fish, sea stars, crustaceans, etc. (Figure 4). The indicator taxa occupied narrow depth ranges with the exception of the sea lilies (Figure 5).

In the upper zone (0–800 m), large rougheye rockfish (*Sebastes aleutianus*; Figure 4a) had the narrowest depth range (2*σ* 102 m; see Figure 5 for max/min depths), were only observed on Union, and peaked in abundance on the summit (truncated upper range). While highly mobile relative to other indicator taxa, rougheye rockfish stay close to the seafloor and have high habitat fidelity (Love, Yoklavich, & Thorsteinson, 2002). Relatively sedentary brittle stars (dominated by *Ophiacantha diplasia* with sparse *Ophiopholis longispina* and *Ophiopholis bakeri*, hereafter “Ophiuroidea”; Figure 4a–c) formed a narrow, near‐continuous, dense mat, at the top of the two shallowest seamounts (2*σ* 194 m). Sparse patches and individual brittle stars were also observed deeper. Two soft corals, both found on the two shallowest seamounts, had the narrowest depth distributions of sessile habitat‐forming groups in the upper zone: the bubblegum coral *Paragorgia* cf. *jamesi* and the bamboo coral *Isidella tentaculum* (2*σ* 304 and 294 m, respectively). Whereas the bubblegum coral was relatively abundant but shorter and simpler (Figure 4b), the bamboo coral was less abundant but larger (some 2 m tall), with complex branching (Figure 4c).

In the middle zone (800–1200 m), the bushy *Chrysopathes speciosa* black coral (Figure 4d) and the vase‐shaped *Pinulasma* n. sp. bugle sponge (Figure 4e), again found on the two shallowest seamounts, had the narrowest depth distributions (2*σ* 242 and 270 m, respectively), adding to the gardens observed at these depths. The abundances of both taxa dipped within these gardens (900–850 m; S5), which were a dense and diverse mixture of indicator taxa and many other corals and sponges (predominantly glass sponge taxa) suggesting interspecific competition for space may play a role in structuring the community (the dip in the black coral was substantial, creating a biomodal distribution; S5). In the lower zone (>1,200 m), the narrowest depth distributions were the small Flabellidae cup coral (2*σ* 176 m; Figure 4f) and the undulated cf *Tretodictyum* n. sp. glass sponge (2*σ* 252 m; Figure 4g). The cup coral was observed on the three shallowest seamounts, while the undulated glass sponge inhabited all four (S5).

These eight taxa had roughly unimodal depth distributions narrower than over two‐thirds of all other annotated organisms (except black coral; S5). For example, these depth specialists inhabited depth ranges three to nine times smaller than the ubiquitous *Florometra serratissima* (2*σ* 952 m; Figure 4h). This sea lily was included in the analysis to represent widely distributed taxa, as dense fields or individuals were observed on all four seamounts, across ~2,150 to 550 m, almost the entire depth range surveyed, with a gap between 1,150 and 700 m (bimodal distribution; S5).

The oxygen and saturation states integrated over the depth bands of these taxa show that current chemical conditions may already cause some organisms stress (Table 2). Furthermore, the oxygen in the water bathing the corals and sponges is declining so rapidly that if trends continue, in the worst‐case scenario, conditions may become fatal within many of their typical life spans (Table 2). Sponges are presumed less sensitive to OA (Bindoff et al., 2019). The absence of this compounding effect may allow for sponge taxa to persist under deoxygenation longer than the corals. The mobility of the sea lily, as well as its broad depth range, may help it endure the changing oxygen conditions. Still, within several generations, if trends continue, oxygen within the entire modern depth range of the sea lily will disappear.

**TABLE 2:** Each indicator taxon, the zone it belongs to, its common and scientific name, number of individuals observed in the 2017 visual surveys, the mean (*µ*) and standard deviation (*σ*) of its depth range (2017), its vertical distribution‐weighted mean oxygen level and trend (1960–2019); its vertical distribution‐weighted mean calcite and aragonite saturation states (Ω_Ca_ and Ω_Ar_) and their trends (1987–2019), as well as relevant life‐history traits (e.g., life span), carbonate mineralogy, hypoxia, and ocean acidification (OA) sensitivity if known. Significant trends are bolded

Zone	Indicator taxon	No. of individuals	Depth *µ*, *σ* (m)	Oxygen *µ* (ml/L) trend (ml/L/century)	Ω_Ca_ *µ* trend (/century)	Ω_Ar_ *µ* trend (/century)	Relevant life‐history traits	Hypoxia sensitivity	Carbonate content, mineralogy/OA sensitivity
<800	Rockfish, *Sebastes aleutianus*	1,258	384, 51	1.8 ± 0.4 0.1 ± 0.5	1.04 ± 0.13 **−0.35 ± 0.18**	0.66 ± 0.08 **−0.22 ± 0.11**	Life span of decades to centuries, oldest to date: 205 years); high habitat and benthic fidelity, depth range 2830–25 m (Love et al., 2002) with a preference for 200–800 m (DFO, 2012)	Sensitive to hypoxia; Not found at ~<1.08 ml/L (Keller et al., 2017)	Little calcium carbonate (aragonite otoliths; Campana, 1999); expect adult fish to be tolerant to OA (regulate internal ion concentrations; Haigh et al., 2015); OA may affect larval success, but no direct evidence for rockfish (Haigh et al., 2015)
<800	Mats of brittle stars, Ophiuroidea	26,699	491, 97	1.16 ± 0.21 −0.3 ± 0.3	0.87 ± 0.09 −0.12 ± 0.13	0.55 ± 0.06 −0.07 ± 0.08	Sedentary suspension detritivores (Lambert & Austin, 2007); likely great energetic demands where gregarious (cf. Metaxas & Giffin, 2004); live up to a decade + (Ravelo, Konar, Bluhm, & Iken, 2017)	Sensitive to hypoxia (Calder‐Potts et al., 2015; Nilsson & Skold, 1996; Riedel et al., 2008)	High‐Mg‐calcite internal structure (Iglikowska et al., 2017); expected to be vulnerable to OA (McClintock et al., 2011); negative OA impacts have been shown (Donachy & Watabe, 1986; Dupont et al., 2008, 2010; Wood et al., 2008)
<800	Bubblegum coral, *Paragorgia* cf. *jamesi*	3,567	582, 152	0.94 ± 0.17 **−1.6 ± 0.9**	0.84 ± 0.08 −0.05 ± 0.13	0.54 ± 0.05 −0.03 ± 0.08	Lives decades (Mortensen & Buhl‐Mortensen, 2005; Sherwood & Edinger, 2009)	No direct O_2_ studies; *Paragorgia* spp. occur in low O_2_ environments (this study; Elliott et al. 2015)	Surface & internal, separate & nearly fused CaCO_3_‐polymorph sclerites (skeletal element) (pers comm Merlin Best), likely high‐Mg‐calcite (Bostock et al., 2015; Le Goff et al., 2017); may be vulnerable to OA (Le Goff et al., 2017), especially exposed sclerites (Gabay, Fine, Barkay, & Benayahu, 2014)
<800	Bamboo coral, *Isidella tentaculum*	431	686, 147	0.63 ± 0.15 **−1.9 ± 0.9**	0.81 ± 0.08 0.02 ± 0.11	0.51 ± 0.05 0.01 ± 0.07	Lives decades to centuries (Andrews et al., 2009)	No direct O_2_ studies; found to be thriving in large colonies in low O_2_ (0.4 ± 0.2 ml/L) (Etnoyer, 2008)	Embedded CaCO_3_‐polymorph sclerites & largely calcareous axis (M. Best, personal communication, July 24, 2020), likely high‐Mg‐calcite and/or aragonite (Bayer & Macintyre, 2001; Thresher et al., 2011); may be vulnerable to OA (Haigh et al., 2015; Rossin, 2018; Thresher et al., 2011), especially exposed parts (Gabay et al., 2014)
Mid	Black coral, *Chrysopathes speciosa*	238	863, 121	0.48 ± 0.17 **−0.75 ± 0.09**	0.78 ± 0.07 0.04 ± 0.1	0.5 ± 0.05 0.03 ± 0.06	Lives decades to centuries (Sherwood & Edinger, 2009; Williams, Risk, Ross, & Sulak, 2006)	No direct O_2_ studies; dense stands have been found in low O_2_ sites (Genin, Dayton, Lonsdale, & Spiess, 1986; Opresko & Genin, 1990)	No calcium carbonate (organic proteins); no direct OA studies (Haigh et al., 2015)
Mid	Bugle sponge, *Pinulasma* n. sp.	1,265	1,026, 135	0.46 ± 0.16 **−0.7 ± 0.13**	0.76 ± 0.07 0.05 ± 0.1	0.48 ± 0.04 0.03 ± 0.06	Lives decades to centuries (Austin, Conway, Barrie, & Krautter, 2007; Leys, Mackie, & Reiswig, 2007)	Sensitive to but tolerant of low O_2_ (Leys & Kahn, 2018); rare at O_2_ ~<1.4–2.1 ml/L (Leys et al., 2004; Whitney et al., 2005)	No calcium carbonate (silicious); presumed insensitive to OA (Bindoff et al. 2019; Conway et al., 2017); no direct OA studies (Haigh et al., 2015)
>1,200	Cup coral, Flabellidae	2,536	1,258, 88	0.51 ± 0.15 **−0.59 ± 0.12**	0.74 ± 0.06 0.02 ± 0.09	0.47 ± 0.04 0.01 ± 0.06	Lives decades (McCulloch et al., 2005)	Sensitive to O_2_ (<3.2 ml/L, Dodds, Roberts, Taylor, & Marubini, 2007; ~<1.5 ml/L, Lunden, McNicholl, Sears, Morrison, & Cordes, 2014)	Largely exposed skeleton (Jantzen & Schmidt, 2013); aragonite (Roberts, Wheeler, Freiwald, & Cairns, 2009); sensitive to Ω (Carreiro‐Silva et al., 2014; Gómez et al., 2018; McCulloch et al., 2012)
>1,200	Undulated glass sponge, cf *Tretodictyum* n. sp.	467	1,630, 126	0.85 ± 0.14 **−0.9 ± 0.3**	0.75 ± 0.06 0.01 ± 0.1	0.48 ± 0.04 0.01 ± 0.06	Lives decades (Austin et al., 2007; Leys et al., 2007)	Sensitive to but tolerant of low O_2_ (Leys & Kahn, 2018); influences population structure (Zeng et al., 2020); rare at O_2_ ~<1.4–2.1 ml/L (Leys et al., 2004; Whitney et al., 2005)	No calcium carbonate (silicious); presumed insensitive to OA (Bindoff et al. 2019; Conway et al., 2017); no direct OA studies (Haigh et al., 2015)
All	Sea lily, *Florometra serratissima*	1,410	1,383, 476, gap: 1150–700	0.81 ± 0.13 **−0.46 ± 0.04**	0.77 ± 0.06 −0.01 ± 0.1	0.49 ± 0.04 0 ± 0.06	Short bursts of locomotion, depth range 11–1,252 m (Lambert & Austin, 2007); lives decades (Murillo et al., 2011); fields are habitat forming (NAFO, 2018)	No direct O_2_ studies; bimodal distribution (this study) suggests low tolerance to low O_2_	High‐Mg‐calcite internal structure (Iglikowska et al., 2017); expected to be vulnerable to OA (McClintock et al., 2011)

While there is no significant long‐term oxygen trend within the rockfish depth range, the movement of the OMZ upper bound likely has and will continue to limit the small habitable area of this hypoxia‐sensitive fish. In 2017, it appears a threshold existed near the OMZ upper bound (*µ*−*σ* = 435 m), which restricted the species to the shallowest part of its preferred depth range (800–200 m, down to 2,830 m; Table 2). Carbonate saturation states are also declining at and above these depths—and may cause direct or indirect (i.e., food web) impacts (Haigh et al., 2015; Hamilton et al., 2017). Brittle stars are vulnerable to OA (Table 2), and the mean carbonate saturation state bathing the ~200 vertical meters of dense brittle star mats, which play a significant role in seamount energy transfer and represent a large proportion of the local benthic productivity and biomass, may be declining.

## 4 DISCUSSION

### 4.1 Climate change impacts on oxygen and calcite in the intermediate and deep NEP

The waters bathing the seamounts are some of the oldest in the global oceans. The majority were last at the surface (i.e., ventilated) allowing oxygen uptake from the atmosphere (and CO_2_ expulsion) ~1,000 years ago (Gebbie & Huybers, 2012), prior to the Anthropocene. Thus, the significant chemical changes that we observe on timescales of decades in the deep water are not expected. Only the tallest summits (Union and Dellwood, Table 1) are shallow enough to experience mixing with the relatively young (5–10 years) thermocline which is ventilated in the Northwest Pacific (NWP; Ueno & Yasuda, 2003; Whitney et al., 2007). Anthropogenic carbon is expected in this water (Carter et al., 2019; Wakita, Watanabe, Murata, Tsurushima, & Honda, 2010) and drives a reduction in Ω (Figure 2). The oxygen trends in this zone (~120–700 m depth in the NEP) are consistent with similar observations on common density surfaces near the ventilation region (Nakanowatari, Ohshima, & Wakatsuchi, 2007; Sasano et al., 2015) and previous local analyses (Crawford & Peña, 2016; Whitney et al., 2007). These are driven at least partly by a reduction in ventilation associated with climate change (Deutsch, Emerson, & Thompson, 2006).

A decade ago, Whitney et al. (2007) found the upper bound of the OMZ to be shoaling, extending the time series to 2019, we do not. This inconsistency highlights the influence of decadal scale variability in the NWP source waters at these depths (evident in Figure 3a, relationship of NWP waters to NEP water properties shown in Crawford & Peña, 2016; Whitney et al., 2007). A longer time series might allow a weak shoaling of the top of the OMZ to emerge from the variability, but changes in the depths of these source waters are also important.

Changes in circulation have forced the downward migration of isopycnals (Figures 2 and 3). It is this migration that brings oxygen to the top of the OMZ and keeps its upper boundary from shoaling, even though oxygen is decreasing on each isopycnal (Figure 3a). This observation is most likely due to circulation changes and possibly flow convergence further offshore in the NEP between ~100 and 500 m (Cummins & Ross, 2020), which may result from changes in wind forcing or distant overturning circulation (Ohshima, Nakanowatari, Riser, Volkov, & Wakatsuchi, 2014).

Slowing down of flow may also be responsible for the changes in oxygen on deep isopycnals. While the deep water is too old to have experienced ventilation during the Anthropocene (mean age 1,000–1,500 years, Gebbie & Huybers, 2012; Matsumoto, 2007), anthropogenically driven changes in circulation (Deutsch, Emerson, & Thompson, 2005; Deutsch et al., 2006; Masuda et al., 2010; Talley et al., 2016) could mean that it is progressively older and has experienced more oxygen consumption (e.g., Lauvset et al., 2020) when it arrives at the seamounts. Trends were weaker on the deeper isopycnals, but they were always negative. Changes at these depths could also result from increased organic matter export (causing oxygen consumption).

### 4.2 Potential loss of ecosystem diversity

The changing chemical environment in the deep NEP will likely cause significant loss of seamount ecosystem diversity. This loss can be investigated using ecosystem classification systems based on environmental variables. For instance, Clark et al. (2011) identified five “biologically meaningful” environmental variables as part of a seamount classification system (biogeographic province, export productivity, summit depth, dissolved oxygen, and proximity) and showed that it gives biologically realistic groupings in comparison with biological data. At present, this scheme shows four classes among the seamounts in our study region (DFO, 2019; S6); Union is the single member of one class (oxic; summit <800 m), Dellwood is in another (hypoxic; summit <800 m), and the UN seamounts are in a third (hypoxic; summit >800 m). Seamount summit oxygen level is a key variable in the scheme, highlighting the potential impact of climate‐driven trends. Projected changes in oxygen at the summits in our study area—from oxic to hypoxic—will cause a loss of the second largest seamount class (oxic; summit >800 m) as it transforms into the hypoxic class at a rate of approximately 1 seamount every 8 years. If the OMZ continues to expand at its present rate, an entire seamount class—representing an ecosystem type—will be lost by 2240, homogenizing the seamount cluster.

Additionally, while taxa were found at similar depth bands across the different seamounts sampled (Figure 5), their absolute and relative abundances were not the same, that is, community structure differed (S5). The loss of large‐scale ecosystem diversity could cause cascading ecological effects. For example, if a class of seamounts supports the source (reproductive) population, seeding nearby seamounts of different classes, then losing a seamount class could lead to the local loss of some taxa.

This classification scheme does not consider the additional stress of OA (DFO, 2019). At its present rate, the rapidly shoaling calcite saturation horizon (equivalently Ω_Ar_ ~ 0.64) will pass the summit of Union seamount by about 2040 (Figures 2 and 5). Union supports a unique offshore shallow‐water community (isolated from the continental slope by 100s of km; Figure 1; Table 1). Additionally, it is home to a number of corals (Figure 4) that already live in low Ω waters and will experience increasing stress in coming years.

### 4.3 Potential impact of chemical trends on NEP seamount communities

While classification schemes are useful for simplifying the description of seamount ecosystems, their thresholds are only meaningful to the bulk assemblage of species (Clark et al., 2011). Individual, or groups of similar, species are influenced by their local environment—even if they are not near an identified threshold. While temperature change drives important changes in Atlantic seamounts (Puerta et al., 2020), temperature trends in the NEP are much weaker (<0.5°C/century below 500 m, Cummins & Ross, 2020), and thus, we have focused on chemical trends. With strong chemical gradients at many depths, the narrow depth distributions of the indicator taxonomic groups identified here (Figures 4 and 5) suggest that their ranges are already limited by their chemical environment (Clark et al., 2010; Wishner et al., 1990). Therefore, they may be particularly sensitive to oxygen and Ω trends, especially if they are long‐lived—as are many of the indicator taxa (see Table 2)—or run out of suitable habitat (e.g., if their ideal chemical zone, e.g., Table 2, passes the summit of the seamount). While variability that overlies the observed trends (Figures 2 and 3) could also play a role in triggering distribution changes, we focus on potential impacts of the long‐term trends (Table 2). This variability is accounted for in the trend uncertainties (S3); if a trend is significant, it has “emerged” from the background variability (in Figure 3, gray bands illustrate typical depth‐dependent within‐year variability). Episodic human activities could also be harmful. Union and Dellwood (<1,500 m) were both previously fished using bottom‐contact gear (DFO, 2019), which will have reduced densities of vulnerable habitat‐forming taxa by scouring (Du Preez, Swan, & Curtis, 2020), but presumably not to the point of changing their overall depth distributions.

#### 4.3.1 Realized niches with respect to oxygen and carbonate saturation states

Although many species are able to tolerate a wide range of environmental conditions (fundamental niche), due to competition or scarcity, they are often found within a much narrower range of conditions (realized niche). With the exception of the sea lilies, the realized niches of all the indicator taxa were narrow, with respect to depth and, consequently, area. The echinoderms, brittle stars (Ophiuroidea) and sea lilies (*F. serratissima*), present a contrast to each other; brittle stars being one of the most narrowly distributed species (found near the upper boundary of the OMZ, oxygen ~1 ml/L; this “edge effect” is observed worldwide, e.g., Levin, 2003; Mosch et al., 2012), while the more mobile sea lilies were most widely distributed even though they showed intolerance to low oxygen (bimodal distribution around the severely hypoxic OMZ core). This distribution suggests the sea lilies are more of a generalist (consistent with paleo records; Lubeseder, 2008) despite both echinoderms avoiding the OMZ (Rogers, 2000).

The other mobile indicator, rougheye rockfish (*S. aleutianus*), has relocated to less preferred habitat in other regions because of shoaling low oxygen water (<1.57 ml/L, Grantham et al., 2004; <0.8 ml/L, Matabos, Tunnicliffe, Juniper, & Dean, 2012; <1.08 ml/L, Table 2). Similarly, on Union seamount, these rockfish were observed inhabiting primarily the upper half of their preferred depth range (Table 2), seemingly to avoid the OMZ. Additionally, the size, weight, and abundance of rockfish are expected to decrease with lower dissolved oxygen levels (Cross, 1987; Keller et al., 2017; Koslow, Goericke, Lara‐Lopez, & Watson, 2011; Vetter & Lynn, 1997). There may also be negative impacts associated with OA (Hamilton et al., 2017).

Carbonate coral distributions are expected to be limited by Ω (Guinotte & Fabry, 2008; Thresher, Tilbrook, Fallon, Wilson, & Adkins, 2011). In the NEP, Ω is low relative to other major ocean basins (Jiang et al., 2015), so we find realized niches at significantly lower Ω than elsewhere. For example, Baco et al. (2017) found Scleractinian reefs of *Desmophyllum pertusum* (also known as *Lophelia pertusa*; Addamo et al., 2016) limited to Ω_Ar_ > 0.71, whereas we found Scleractinian cup corals (Flabellidae) occupying depths with much lower Ω_Ar_ (0.47; Table 2). Similarly, we find cup corals at low oxygen—within the hypoxic zone (0.51 ml/L)—even though oxygen is considered a limiting factor in the distribution of *Desmophyllum* corals (Table 2) and most *D. pertusum* reefs occur in oxic water (3–5 ml/L, Freiwald, 2002; 5–7 ml/L, Wisshak, Freiwald, Lundälv, & Gektidis, 2005). Similar to the recently discovered *D. pertusum* reefs in the Southeast Atlantic, an extensive food supply may be increasing our corals capability to adapt to extreme hypoxic conditions (e.g., Hebbeln et al., 2020). Both the bamboo coral and bubblegum coral were found in waters with low oxygen (*Isidella* spp.: 0.63 ml/L, this study and 0.4 ± 0.2 ml/L, Etnoyer, 2008; *Paragorgia* spp.: 0.94 ml/L, this study and <2.6 ml/L, Elliott, McLetchie, Kelley, & Wagner, 2015) and low Ω (*Isidella* spp.: Ω_Ar_ = 0.51, this study and <0.7, Thresher et al., 2011; *Paragorgia* spp.: Ω_Ar_ = 0.54, this study and <0.9, Bostock et al., 2015) consistent with previous studies. Being made of organic proteins, the niche of the black coral (*C. speciose*) is expected to be the least influenced by Ω (Table 2). Chu et al. (2019) show that black coral's habitat influences do not appear to include inorganic carbon or oxygen.

Like black coral, the glass sponges, both bugle (*Pinulasma* n. sp.) and undulated glass (cf *Tretodictyum* n. sp.), are not composed of carbonate, and therefore, Ω is not expected to limit their niches (Table 2). However, oxygen levels below 1 ml/L may pose physiological limitations for glass sponges, and they are already rare when oxygen <1.4–2.1 ml/L and silicic acid, the key building block for glass sponges, is abundant (Leys et al., 2004; Whitney et al., 2005). Glass sponge reefs were present over 500 million years ago (Gehling & Rigby, 1996), implying enormous resiliency to extreme conditions in both oxygen and Ω (Bindoff et al., 2019), which they will need in the coming centuries.

Models predict that North Atlantic seamounts may act as temporary refugia from deep OA for cold water corals (Tittensor, Baco, Hall‐Spencer, Orr, & Rogers, 2010), but that climate‐induced changes will ultimately reduce suitable habitat for corals (up to 100% reduction) and fishes, by 2100 (Morato et al., 2020). While Ban, Alidina, Okey, Gregg, and Ban (2016) have suggested that some shallow NEP seamounts (including Union) may similarly provide future climate change refugia, the long‐term chemical trends documented here show that this result is unlikely. Similar trends, as well as an increase in temperature and a substantive decrease in particulate organic carbon flux to the seafloor, are predicted by global models for the Pacific (Sweetman et al., 2017) and North Pacific High Seas (FAO, 2018).

#### 4.3.2 Mortalities associated with low oxygen

Although some organisms are adapted to low oxygen conditions, all need oxygen for respiration and if deprived for too long, mortality will result, even for the resilient glass sponges (Leys et al., 2004). Our data show visual evidence that bamboo coral, living near the upper bound of the OMZ and so generally tolerant of hypoxia (Etnoyer, 2008) and oxygen variability (Figure 2), likely experienced one or more episodic mortality events. Starting at 650–700 m, there is a sharp decline in living bamboo coral abundance, with very few found within the severely hypoxic OMZ core and none below (S5). Almost a quarter of the corals between 650 and 700 m were erect intact skeletons, bare of living tissue. This result indicates environmental stress, not a fishing‐related impact (Du Preez et al., 2020), perhaps by an incursion of low oxygen water, a fluctuation in Ω, disease, or even damage to flesh by predation exposing calcareous skeletal elements. Additive or synergistic effects of multiple stressors may increase the cumulative stress and overall susceptibility (e.g., Steckbauer, Klein, & Duarte, 2020). With extremely slow recolonization expectations and growth rates of approximately 1 cm/year (Table 2; Andrews, Stone, Lundstrom, & DeVogelaere, 2009), these 2 m coral stands will take centuries or longer to recover, and only if conditions remain suitable.

While sessile organisms are most at risk to low oxygen, mobile species, especially “mountaintop” species, can indirectly suffer a mortality event if pushed off (e.g., butterfly extirpation on warming mountain peaks, Miller, Janzen, & Hallwachs, 2007). Low oxygen excursions near the edge of their distribution may “push” rougheye rockfish off the summit of Union seamount (Figure 5: truncated upper depth distribution) and may have already caused a local mortality event of rougheye on Dellwood seamount, similar to those associated with upwelling on the Oregon shelf (Grantham et al., 2004). For decades, Dellwood supported an abundant population of rougheye (DFO, 2019) but, in 2017 (our study) and 2019 (unpublished data), not one rougheye was observed. Like mountaintop species, seamount summits are vulnerable to changing conditions and experience low recolonization potential. Thus, the offshore population of this species of conservation concern and commercial value (DFO, 2012) is already under threat. As one of the longest lived fish species (up to 205 years old; Table 2), rougheye rockfish population recovery could take decades to centuries.

#### 4.3.3 Difficulty in forming structural elements under ocean acidification

A first‐order effect of OA is the increased energetic cost to form carbonate body parts (Spalding et al., 2017), such as sclerites in corals. Many organisms are successful in low Ω waters (Figures 2 and 5), but energetic costs will increase as Ω decreases under OA, leaving less energy for other functions like reproduction (Kroeker et al., 2013). For example, some echinoderm species have shown slowed arm regrowth (Donachy & Watabe, 1986) and muscle wastage (Wood, Spicer, & Widdicombe, 2008) under low Ω. Brittle stars contribute significantly to local seamount productivity and provide community structure. Their mean depth is just below the depth range of significant Ω trends; some portion is likely experiencing decreasing Ω (Table 2; S5). Brittle stars have strong but highly soluble high‐magnesium‐calcite structures, so they already require significant energy to calcify (Table 2). Arm regrowth is frequent (Donachy & Watabe, 1986); thus, the stress of declining Ω threatens what may already be a delicate balance for this taxon. Sea lily structures are more soluble than those of brittle stars (Iglikowska, Najorka, Voronkov, Chełchowski, & Kukliński, 2017); however, sea lilies calcify less frequently and are more fleshy (P. Lambert, personal communication, November 11, 2019), with organic coatings isolating body parts from seawater.

Organic coverings are likely key to the success of several of our indicator taxa. As with the brittle stars, parts of other echinoderms (McClintock et al., 2011 and references within) and bubblegum and bamboo corals (Bayer & Macintyre, 2001; Bostock et al., 2015; Haigh et al., 2015; Le Goff et al., 2017; Thresher et al., 2011) are composed of high‐magnesium‐calcite. Bubblegum corals (*Pargagorgia* spp.) in Antarctica tolerate low Ω (Ω_Ar_ ~ 0.7) due in large part to the fact that the minerals are covered with organic material and thus not directly exposed to seawater (Bostock et al., 2015). Not all coral skeletal elements are internal—surface and tentacular sclerites, bare holdfasts, and axes can all be exposed and so bathed in seawater (observed in images and collections; M. Best, personal communication, July 24, 2020). On the bamboo coral segments of the axis were often not covered by organic material, yet this coral was observed in significant numbers in more corrosive waters than bubblegum coral in our study area (Figures 2 and 5), highlighting the complexity of factors defining realized niches. Cup corals have an aragonitic exoskeleton most of which is directly bathed in seawater (Table 2; Jantzen & Schmidt, 2013). Survival in the low Ω waters on NEP seamounts must come at a significant energetic cost (Carreiro‐Silva et al., 2014; Gómez, Wickes, Deegan, Etnoyer, & Cordes, 2018).

Even for species that contain no or little carbonate, if they are living (and breathing) animals, they must maintain ion transport over membranes (which can be impacted by pH) and must rid themselves of CO_2,_ so there will likely be OA impacts on chemical regulation in all animals (Claiborne et al., 2002). Ion transport difficulty may be a factor in the observation that high CO_2_ levels (1,125 µatm) induced anxiety relative to ambient CO_2_ levels (483 µatm) in rockfish, as observed through scototaxic (light/dark preference) testing (Hamilton, Holcombe, & Tresguerres, 2014). There is also some evidence suggesting that OA impacts will be more severe in larval stages (e.g., Hurst et al., 2019).

#### 4.3.4 Ocean acidification impacts on larvae and dispersal

OA impacts many aspects of reproduction; spawning, larval development, and juvenile mortality. For example, *Primnoa pacifica* (same suborder as the bamboo coral; inhabits the study seamounts; data not shown) experienced reduced spawning in low pH (pH = 7.55; Rossin, 2018). The keystone brittle star *Ophiothrix fragilis* is especially sensitive to OA, with 100% mortality of larvae with a modest increase in acidity (pH = 7.9; Dupont, Havenhand, Thorndyke, Peck, & Thorndyke, 2008). In a recent seascape genetics study, water chemistry in the North Atlantic was identified as a driver of genetic variability in cold water coral and sponge populations (Zeng, Rowden, Clark, & Gardner, 2020), indicating changes in Ω (and oxygen) over space and time could influence population connectivity and structure by creating or removing genetic barriers.

If detrimental effects on the larval stages of our seamount fauna are significant, dispersal of species within and between the seamounts could be impacted. Larval life span is integral in seamount community structure, determining the subset of species that inhabit and dominate a seamount (Parker & Tunnicliffe, 1994). For several species, we observed high abundance on some seamounts and low abundance on others (S5), which could indicate a strong population on one seamount is seeding the smaller populations on others. If OA reduces fecundity, shortens larval life spans, or weakens the viability of settled larvae, these smaller satellite populations may become vulnerable, even disappear. Increasing OA could restructure populations and communities on individual seamounts and within a seamount network through larval impacts—even if adult populations were resistant to changes in Ω and pH.

#### 4.3.5 Behavioral and food supply changes associated with low oxygen or low Ω

Low oxygen or low Ω triggered behavior or metabolic changes could impact the ability to thrive in a habitat. Brittle stars become less mobile and more vulnerable to predation under low oxygen, leading to shifts in trophic interactions (*Ophiothrix quinquemaculata*, Riedel, Stachowitsch, & Zuschin, 2008). Additionally, hypoxic treatments of the brittle star *Amphiura filiformis* have shown impacts on metabolism and reproduction (Calder‐Potts, Spicer, Calosi, Findlay, & Widdicombe, 2015) and reduced arm growth and disturbance of spawning (Nilsson & Sköld, 1996). Behavioral change under low oxygen has been shown for glass sponges; they reduce (energetically expensive) filtering (i.e., feeding) rates (Leys & Kahn, 2018). However, they can likely survive long periods of low oxygen if currents are strong enough because they take advantage of currents for feeding (Leys et al., 2011). OA may also impact membrane pumps of glass sponges, reducing feeding efficiency (Conway, Whitney, Leys, Barrie, & Krautter, 2017).

The biggest effect of deoxygenation and OA may come through food supply. As the OMZ expands—particularly the upper boundary—the depth to which zooplankton and myctophids migrate daily may shoal as they avoid hypoxic waters (Bianchi, Galbraith, Carozza, Mislan, & Stock, 2013). This daily migration directly and indirectly delivers organic carbon to depth (Steinberg et al., 2000). Hypoxic conditions have been shown to affect the daytime zooplankton depth in many locations (Devol, 1981; Netburn & Koslow, 2015). This avoidance of a rising OMZ boundary would have cascading effects on the already food‐limited systems below (compounding any coincident increase in energy demand). The resident detritus‐based food web on seamounts is already considered fragile, with many organisms near the margin of energetic sustainability, and has also taken a long time to develop (Pitcher & Bulman, 2007). Additionally, OA may increase primary production slightly (Eberlein et al., 2017; Haigh et al., 2015) but decrease its nutritive value causing cascading negative impacts to secondary producers (Riebesell et al., 2007; Rossoll et al., 2012). Furthermore, crustaceans, especially copepods, dominate the zooplankton in the region (Mackas & Tsuda, 1999), are a key food source for the seamounts (e.g., Dower & Mackas, 1996), and may be vulnerable to OA (Cripps, Lindeque, & Flynn, 2014).

### 4.4 Implications for seamount management

States and organizations are moving to protect and monitor an unprecedented amount of the world's oceans. Many seamounts, including those in and around our study area, are identified as marine conservation targets and are within proposed marine protected areas (MPAs), whose objectives are to safeguard the wealth of marine animals living on the seamounts from harmful human activities (e.g., the proposed Offshore Pacific MPA, DFO, 2019; Figure 1). At the same time, the area of OMZs is expanding worldwide (Breitburg et al., 2018). Our findings show that benthic ecosystems and species in our NEP seamounts region are experiencing changes in their chemical environment. These deep‐water chemical stressors have a remote source and cannot be prevented by any spatial management plan. However, impacts from these large‐scale stressors would be compounded by others that the MPA can mitigate—eliminating the controllable impacts may allow these ecosystems to withstand climate‐forcing changes better or for longer (Puerta et al., 2020).

Our documentation of relatively rapid large‐scale chemical changes and their potential ecological effects suggests an impending threat to seamount ecosystems within and beyond the study area and highlights the importance of precautionary management and conservation efforts. For instance, seamount chains to the north (the SGaan Kinghlas–Bowie seamounts) and in the international waters of the NEP, which border the southwestern edge of the proposed MPA (Figure 1) are all bathed in the OMZ (Curtis et al., 2015; Gale et al., 2017). While the former is protected from direct harmful human activities (SGaan Kinghlas–Bowie Marine Protected Area), the seamounts in international waters still experience bottom‐contact fishing (North Pacific Fisheries Commission [NPFC], 2019) and may have to contend with deep‐ocean mining in the future under the International Seabed Authority (i.e., these seamounts are located in a global permissive area for cobalt‐rich crust development; Hein, Mizell, Koschinsky, & Conrad, 2013).

With seamounts, seamount‐like features (Yesson et al., 2020), expanding OMZs (Breitburg et al., 2018), and other climate‐forcing changes (e.g., Sweetman et al., 2017) distributed throughout the world's oceans, this study highlights the global importance of mitigating direct human impacts as species continue to suffer environmental changes that are beyond our control in the immediate future, and the need for climate‐informed ecosystem‐based management, monitoring, and protection, including built‐in resilience to uncertainties or projected changes in ocean conditions over multiple timescales (e.g., Gehlen et al., 2015).

## 5 SUMMARY

Benthic communities on NEP seamounts are experiencing significant long‐term changes in their chemical environment; the OMZ is expanding, deep oxygen levels are decreasing (15% since 1960), and waters above the OMZ are becoming more corrosive. These changes are caused by global reduction in ventilation, anthropogenic carbon accumulation, and, likely, a slowdown in deep large‐scale circulation. Many organisms already occupy narrow depth zones at or beyond their optimal oxygen limits. If these chemical trends continue as they have over the last three to six decades, the seamount ecosystems and their associated animals will be severely impacted. The study taxa are a subset of indicators that represent four phyla and have varying depth distributions, ecological roles, and life‐history traits. Despite the biological variety and distribution across almost two vertical kilometers, evidence shows all of these taxa are vulnerable to the observed chemical trends—results that are likely applicable to many seamount taxa, even the generalists. Some populations are likely already experiencing impacts, while others may be more resilient (potentially glass sponges and sea lilies). Regardless, we predict that within the next century, the indicator taxa, many cold water corals, will suffer the compounding effects of, among other things, impacts to their distribution, respiration, metabolism, growth, dissolution, feeding, behavior, reproduction, and, ultimately, their survival or extirpation in our changing oceans. These trends and ecological implications warrant moving beyond the precautionary management and protection approaches toward plans that support and build resilience for future large‐scale declines in ocean ecosystem health.

## Supporting information

Supplementary Material

Supplementary Material

## Data Availability

The chemical and physical data that support the findings of this study are openly available from the Line P data archive at https://www.waterproperties.ca/linep/cruises.php. Additionally, DIC data collected prior to 2016 are publicly available through the Ocean Carbon Data System (OCADS): https://data.nodc.noaa.gov/cgi‐bin/iso?id=gov.noaa.nodc:0110260 (DIC and TA data collected after 2016 will also be archived with OCADS). The benthic survey data that support the findings of this study are available in the supplementary material of this article.
