# Supplementary material for: Rapid deep ocean deoxygenation and acidification threaten life on Northeast Pacific seamounts
Source: Glob Chang Biol. 2020 Sep 14;26(11):6424–44. doi: 10.1111/gcb.15307 (PMC7693292; doi:10.1111/gcb.15307)
Supplement: Supplementary file 1 — Supplementary Material [file GCB-26-6424-s001.pdf]

Rapid deep ocean deoxygenation and acidification threaten life on Northeast Pacific seamounts

Supporting Information

Authors: Tetjana Ross, Cherisse Du Preez and Debby Ianson
Affiliation: Institute of Ocean Sciences, Fisheries and Oceans Canada, Sidney, BC, Canada

S1. Determination of the carbonate system

Precipitation or dissolution of a given calcium carbonate polymorph (such as aragonite or calcite) proceeds abiogenically based on whether the carbonate saturation state (Ω):

$$\Omega = \frac{[CO_3^{2-}][Ca^{2+}]}{\kappa'_{sp}},$$

is >1 (precipitation) or <1 (dissolution), where the stoichiometric solubility constant κ'_{sp} is polymorph specific (Mucci 1983). We determined the carbonate system and solved for the aragonite and calcite saturation states (Ω_{Ar} and Ω_{Ca}) based on the pair of (observed) dissolved inorganic carbon (DIC) and (empirically-derived) total alkalinity (TA: S2) along with the observed phosphate and silicic acid concentrations, pressure, temperature, and salinity values using the Matlab version of CO2SYS 2.1 (Lewis and Wallace 1998; Van Heuven et al 2011). The DIC data were adjusted based on the output of the PACIFICA (PACIFic ocean Interior CArbon) Data Synthesis Project (Suzuki et al 2013) for cruises where adjustments were available. The carbonate dissociation constants used were those from Lueker et al (2000), the bisulfate ion dissociation constant of Dickson (1990), and total borate concentration equations from Uppström (1974). The concentration of calcium ion ($[Ca^{2+}]$) was calculated from salinity (Riley and Tongudai, 1967). Certified Reference Materials were used in the analysis of DIC from 1990 onward. There are only 6 profiles of DIC used in this analysis prior to 1990 and the Omega horizons calculated from them cluster tightly around the trend lines showing no bias (Figure 2), thus the full time-series has been retained.

Additionally, the routines described in Orr et al (2018) were used to combine and propagate the analytical uncertainties in the observations (DIC, ± 2 µmol/kg; temperature, ± 0.002 ºC; salinity, ± 0.003 psu; silicic acid, ± 1.6 µmol/kg; phosphate, ± 0.03 µmol/kg), uncertainty in empirically derived TA (± 9, 10, or 7 µmol/kg, S2), and the uncertainties in the constants to determine the overall uncertainties in Ω (see S3 for more details on how this uncertainty was propagated into uncertainties in horizons and trends).

S2. Total Alkalinity-Salinity relationships

Like salinity, dilution (precipitation and evaporation) exerts the primary control over variations in TA (Millero et al 1998), making linear approximations in TA as a function of salinity possible. Regional variation is expected (e.g. Ianson et al. 2003; Lee et al. 2006; Fry et al. 2016), thus we developed predictive TA-Salinity relationships using the recent TA observations from the Line P DIC data-set.

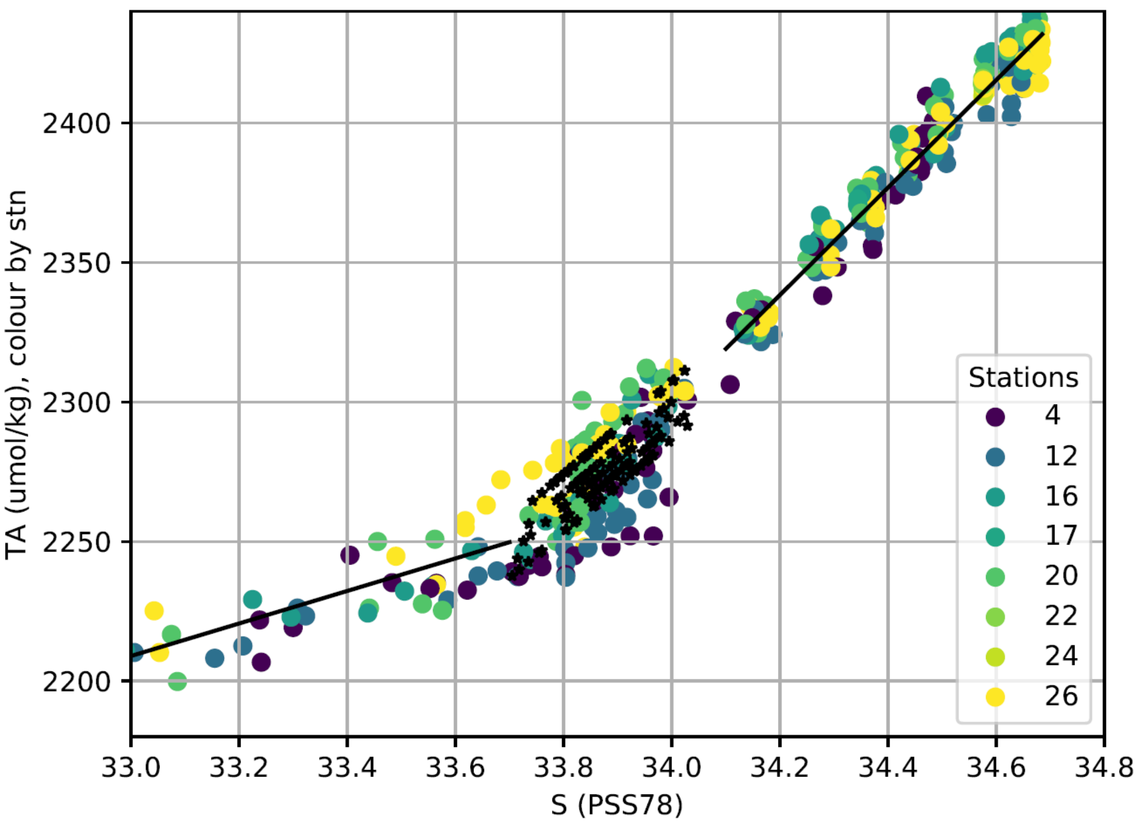

Figure S1: Total alkalinity as a function of Salinity (practical scale PSS78) colored by station along Line P (Figure 1). The three empirical TA-relationships are shown in black; linear f(Salinity) in the surface boundary layer (Salinity ≤ 33.7); black stars indicate predicted TA f(Salinity, longitude) corresponding to each observed TA in the mid-depth (33.7 < Salinity ≤ 34.1) zone; linear f(Salinity) in the deep zone (Salinity > 34.1).

In our study region there are two strongly linear regions in TA-Salinity; in the surface boundary layer and in the deep (Fig S1). These zones are separated by a complex intermediate-depth zone in which water mass mixing and calcium carbonate ($CaCO_3$) dissolution dominate variations in TA-Salinity, rather than simple dilution. We use TA data from recent Line P surveys, seaward of and including P04 (Figure 1) in which the TA replication is within analytical expectations (± 4 μmol/kg, Dickson et al 2007) and there are few, if any, visible outliers (World Ocean Circulation Experiment - WOCE - standard Quality flag 3 or 4). Data from 5 cruises were used in the following analysis: 8-22 Feb and 16-31 Aug, 2016; 5-21 Feb and 4-20 Jun, 2017; and 5-23 Feb, 2019.

Near surface relationship

The upper zone extended to a salinity of 33.7 (Fig S1), which corresponds to roughly the depth of winter mixing (~150 m), and therefore this zone is above the peaks of the seamounts in this study. The upper zone has a simple dilution relationship,

TA = 58.29 * Salinity + 285.42; R^2=0.92, N=98,

which results in an uncertainty of ± 7 µmol/kg. Conservatively, we used an uncertainty of ± 9 µmol/kg for the near surface relationship.

Only winter data were used for the relationship in this zone to avoid possible influence of biological calcification during the productive summer season. Calcifying phytoplankton are common along Line P (Lipsen et al 2007).

Deep relationship

Below a salinity of 34.1, which occurs roughly at a depth of 500 m in our study region, the water is older and has been influenced by remineralisation of organic matter and dissolution of $CaCO_3$. TA-Salinity is also linear in this depth range but has a much steeper slope than in the surface boundary layer,

TA = 192.7 * Salinity - 4252.4; R^2=0.965 N=244,

which results in an uncertainty of ± 7 µmol/kg.

This relationship was developed from all the data available in this salinity range (Fig S1). Nearly all of the indicator taxa in this study occur in this region of TA-Salinity space (with the exception of Rockfish) and many of them occur solely in this region (i.e. deeper than about 500 m).

Mid-depth relationship

Between the upper and deep zones (33.7 < Salinity ≤ 34.1), at intermediate depths (roughly 150-450 m) in the permanent halocline and thermocline, TA is not as tightly related to salinity (Fig S1). The calcite saturation horizon is found in this region and, as this is the least soluble polymorph, any falling biogenic calcium carbonate is likely to dissolve, which may create sporadic and localized TA sources (e.g. Feely et al 2004). In addition, water masses with different end-members meet in this zone. The proportion of water masses varies in space and time, the more western stations having higher TA at the same salinity (Fig S1; Fry et al 2016).

Because we are interested in accurately representing the depth of the Ω_{Ca}=1 and Ω_{Ar}=0.7 horizons, instead of simply joining the deep and upper relationships (which leads to higher uncertainties), we developed a TA relationship dependent on both salinity and longitude (expressed as decimal degrees East) in this zone,

TA = 166.0 * Salinity - 1.121 * longitude – 3500 ; R^2=0.65; N=143,

which results in an uncertainty of ± 10 µmol/kg (the standard deviation of the residuals for stations P12 and P16). In this multi-parameter relationship, salinity explains 76% of the variability and longitude the remainder. As in the deep zone, all data were used (Fig S1).

This three part relationship was used to calculate TA from salinity at all depths for each salinity profile.

S3. Uncertainty and its propagation

Given the available data for oxygen and DIC/TA, the uncertainties in oxygen and Ω levels (and their trends) reflect more the natural temporal and spatial variability for oxygen (with better temporal and spatial coverage) and more the analytical uncertainties in the input parameters used to determine Ω.

Oxygen uncertainty

Combining interpolated bottle data and SBE 43 oxygen sensor data calibrated to these bottle data (SBE 2012), there were anywhere from 1 to 56 oxygen profiles collected each year at each station, on average 3 (from each of the spring, summer and winter Line P cruises) per station and anywhere from 1 to all stations were sampled. Thus in a given year between 7 and 122 oxygen profiles were averaged to compute the study-area mean oxygen profile specific to that year (OMZ horizons were calculated for each profile and then averaged). In the early part of the record, the spatial resolution was poor (1-4 stations sampled in year), but cruises were more frequent, while in the last decade, cruises were only 3 times per year, but all stations were sampled and often more than one cast done per station (including the fully resolved sensor data). Thus, the standard deviations of the annual-average study-area mean oxygen and OMZ boundaries were dominated by intra-annual temporal variability (and inter-sample precision) in the early part of the record and seasonal and spatial variability in the latter part of the record. This variability was larger than the presumed analytical uncertainty (e.g. from Whitney et al 2007), so the analytical uncertainty was not explicitly considered for oxygen.

Ω uncertainty

For the carbon data, there were far fewer observations and the analytical uncertainty was much larger, so the uncertainties were treated differently. All the carbon data are presented by cruise. No yearly averages were taken. The same pooling of profiles was done, but as only two stations were used in the spatial average there were generally only 1 or 2 DIC profiles in the study region for each cruise, so the between-profile standard deviations did little to constrain the uncertainty. Instead, we propagated all the analytical uncertainties (detailed in S1 and S2) in the calculation of Ω (S1) using the Orr et al (2018) method. To calculate the uncertainties in the depth horizons from these uncertainties in Ω (ΔΩ), we constructed two “worst-case” profiles (Ω+ΔΩ; Ω-ΔΩ) and calculated the horizons for each scenario in addition to the mean Ω profile. The final uncertainty associated with the given horizon was then half of the difference between the two “worst-case” horizon depths. If the horizon occurred at a depth at which the vertical gradient in Ω is small, a small ΔΩ can lead to a large uncertainty in the horizon, so the ΔΩ translate non-linearly into horizon uncertainties.

Trend uncertainty

The uncertainties for oxygen and Ω described above were used to weight the least squares linear fit for each trend reported (weighting accounts for the relative uncertainty, which was variable over time). The first type of uncertainty in the trend, due to interannual variability around the multi-decadal trend line, was estimated using bootstrapping (Efron and Gong 1983). Additionally, a second type of trend uncertainty was calculated using standard error propagation methods (i.e. for $y=a*t$, $(\Delta a)^2=1/(\Sigma(t_i^2/\Delta y_i)^2$, in order to account for the impact of the absolute uncertainty in the oxygen and Ω data on trend uncertainty. These two uncertainties were combined to give the total trend uncertainties reported in the manuscript (e.g. Figure 2; Table 2).

S4. Aragonite saturation horizon

The aragonite saturation horizon occurs at salinities near the transition from the near-surface TA-Salinity relationship and the mid-depth salinity/longitude relationship and therefore its estimation is sensitive to the exact choice of salinity bounding the near-surface and mid-depth regions and can cause inconsistencies at the matching point. Thus, we chose to use only the near-surface TA-Salinity relationship (S2) when estimating the aragonite saturation horizons (Fig S2). At the higher salinities (at the top of and just inside the mid-depth zone) the horizon estimates may be biased higher in the water column (as the true TA at mid-depth is generally higher than the near-surface estimate based on in-situ salinity).

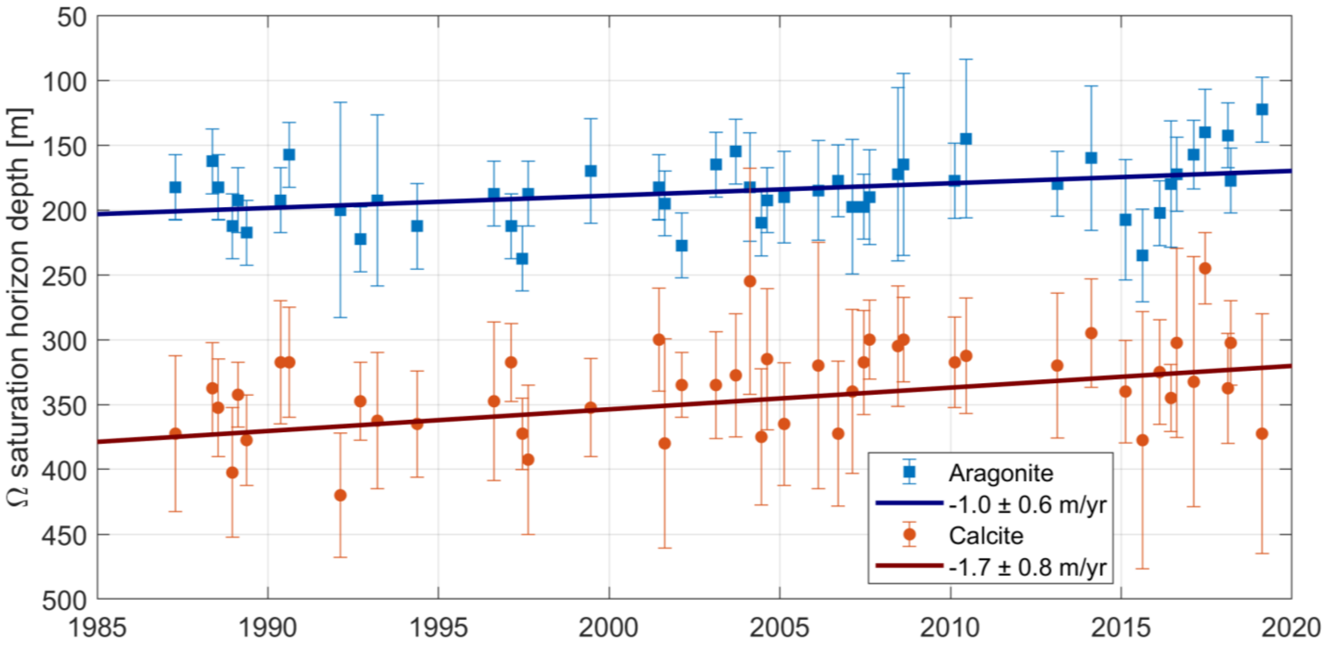

Figure S2: Timeseries of calculated Aragonite and Calcite saturation horizons and their estimated trend lines. The uncertainties stated for the trends are bootstrapped 68% confidence intervals combined with propagated uncertainties (i.e. the uncertainties shown with the error bars).

The estimated shoaling rate of the aragonite saturation horizon, -1.0±0.6 m/yr (Fig S2), is consistent with previous observations. Feely et al (2012) observed aragonite saturation horizons shoaling at a rate of -0.8 m/yr along a hydrographic line about 1400 km west of our study region.

The shoaling rate of the calcite saturation horizon is approximately twice that of the aragonite saturation horizon. The calcite saturation horizon occurs in the middle of the mid-depth salinity range in our data. The calcite horizon is currently well below the depth of the deepest (winter) annual surface boundary layer (approximately the depth of the permanent pycnocline ~120-150 m). The aragonite saturation horizon is already quite close to this depth, its shoaling rate is likely constrained by occasional interactions with the surface boundary layer.

S5. Community structure

While we found taxa at similar depth ranges across the four seamounts surveyed, our data show the benthic community structure differed between seamounts (i.e. there were differences in what taxa were present and in what abundances; presence-absence data are summarized in Table S1). The shallowest seamount, Union, was found to support all nine indicator taxa. The next shallowest seamount, Dellwood, supported all but one taxon (no rockfish observed). Summiting at much deeper depths, UN 16 and UN 18 supported fewer indicator taxa: UN 16 supported the cup coral, glass sponge, and sea lily while UN 18 supported only the latter two. Intuitively, differences in taxa presence-absence correspond to how much, if any, of the seamounts protrude into their depth range. All taxa were found on all seamounts with habitat (seafloor) in their depth range (mean ±1 standard deviation) with the exception of the bugle sponge which was not observed on UN 16.

Table S1: The absolute abundance, for each indicator taxon, on the four seamounts. Where 0 is interpreted as either absent or so rare that detection is outside the scope of this study.

Indicator taxa	Union	Dellwood	UN 16	UN 18	Total
Rockfish, *Sebastes aleutianus*	1258	0	0	0	1258
Mats of brittle stars, Ophiuroidea	25,392	1,307	0	0	26,699
Bubblegum coral, *Paragorgia* cf *jamesi*	2313	1254	0	0	3,567
Bamboo coral, *Isidella tentaculum*	257	174	0	0	431
Black coral, *Chrysopathes speciosa*	6	232	0	0	238
Bugle sponge, *Pinulasma* n. sp.	1194	71	0	0	1,265
Cup coral, Flabellidae	626	794	1116	0	2,536
Undulated glass sponge, cf *Tretodictyum* n. sp.	10	9	446	2	467
Sea lily, *Florometra serratissima*	420	526	223	241	1,410

The depth profiles of the relative abundances of the indicator taxa shown in Fig S3 further illustrate the differences in the benthic community structure between seamounts. An optimal habitat (supporting high abundances) likely depends on a complex combination of interrelated environmental and biological factors (Pitcher and Bulman et al 2007; beyond the scope of this study). One such factor may be the proximity of the depth ranges of the taxa to the depth of the seamount summit. This, in turn, may relate to the amplified food delivery, nutrient upwelling, resuspension of detritus, increased vertical mixing, etc. caused by bottom flow acceleration experienced in the depth range of the seamount summit (Pitcher and Bulman et al 2007). Proximity to the summit may explain why deeper taxa are more abundant on seamounts with deeper summits (e.g. the black coral is relatively more abundant on Dellwood than Union; the cup coral and undulated sponge are more abundant on UN 16 than Union and Dellwood).

In addition to illustrating the differences in community structure between seamounts, Fig S3 clearly shows (and emphasizes) a number of depth-distribution characteristics discussed in this study, including the width of the depth-distributions (narrow except for the sea lily), the truncated upper depth-distribution of the rockfish (possible ‘mountaintop’ species), and the 900-850 m dip of the bugle sponge and bimodal

depth-distribution of the black coral (possible interspecific competition) and the bimodal depth-distribution of the sea lily (possible avoidance of low oxygen).

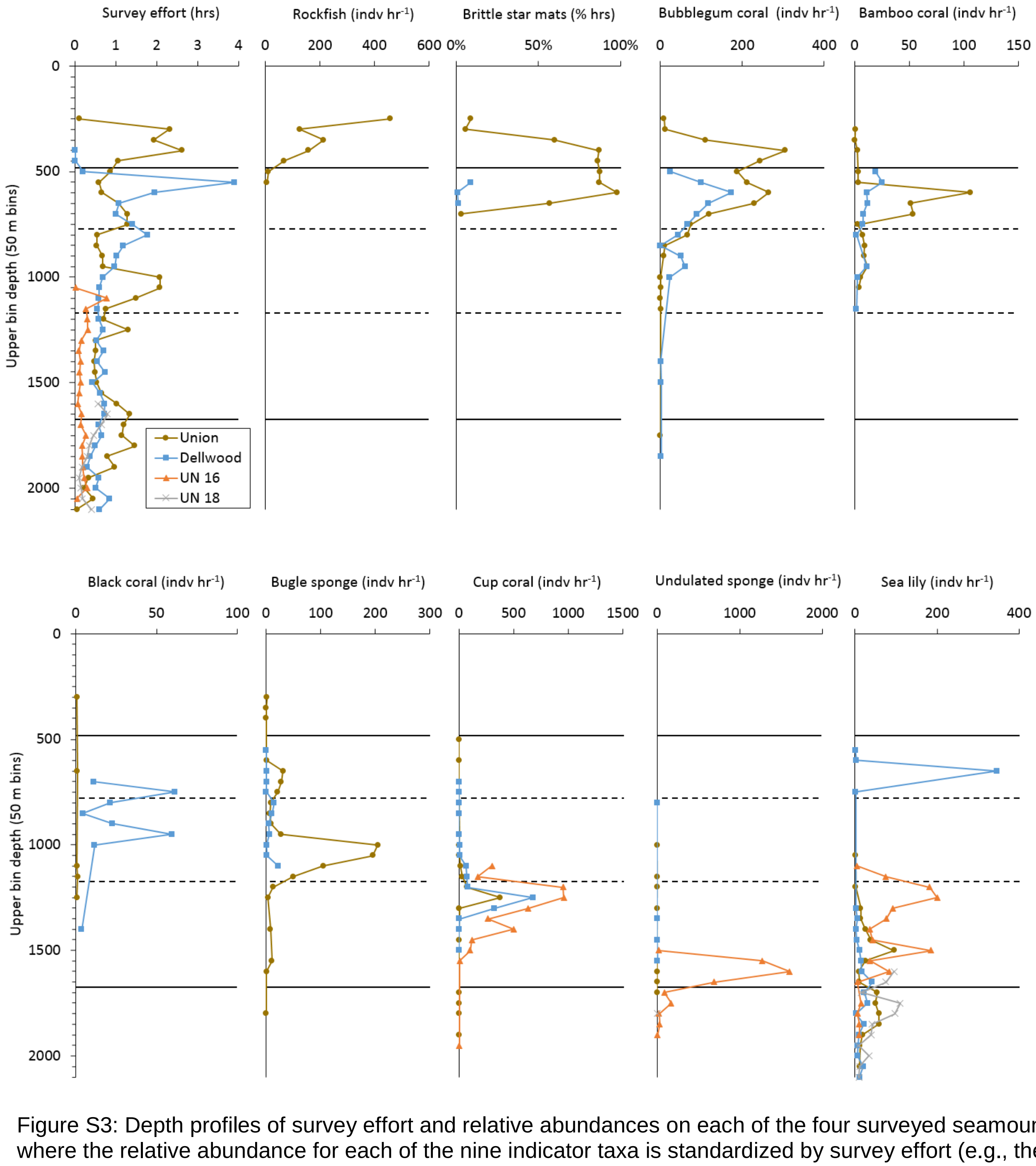

Figure S3: Depth profiles of survey effort and relative abundances on each of the four surveyed seamounts, where the relative abundance for each of the nine indicator taxa is standardized by survey effort (e.g., the number of individuals counted per hour of benthic visual survey video annotated). Depth is binned in 50 m increments, with the upper depth of the interval plotted (i.e., 500 m = 500-550 m interval). The shallowest bins surveyed for each seamount are: Union 250-300 m, Dellwood 400-450 m, UN 16 1050-1100 m, and UN 18 1600-1650 m. All seamounts were surveyed to at least 2100 m depth. The solid and dashed horizontal lines delineate the regions between 480-1700 m and 800-1200 m which correspond to where oxygen is <1.0 ml/l and <0.5 ml/l). The oxygen data is depth-specific and so is plotted between bins here (e.g. the 800 m boundary, upper dashed line, is plotted between 750-800 and 800-850 m).

S6. Seamount classification results

The impact that these trends could have on the ecosystem diversity of seamounts in the AOI is illustrated in Fig S4. Using the seamount classification scheme of Clark et al (2011; DFO 2019) and assuming a sustained linear rate of change in the depth of the OMZ boundaries (0 for upper boundary and 3.1 m/yr for lower boundary) and the observed or predicted summit depths of the 46 seamounts in the study region, each seamount was classified at each yearly time step and the resulting timeseries are shown in Fig S4. The biggest change over time is found in the deeper seamount classes (summits below 800 m), 1 and 2, with the population of Class 1 changing from about half of the seamounts in 1960 to less than one third in 2019. If the deepening trend of the bottom of the OMZ boundary continues at the same rate throughout the 21st century, by the end of the century there will only be 8 seamounts remaining in Class 1. Membership in classes 3 and 4, however, is small and doesn't change over time. This result is robust even with minor shoaling of upper OMZ boundary because the summit of Union (285 m) is well above the present mean depth of the OMZ. However, the summit of Union seamount is predicted to cross the calcite saturation horizon sometime in this decade (the line thickness for Class 4 in Fig S4 indicates whether Union has $\Omega_{Ca} \geq 1$).

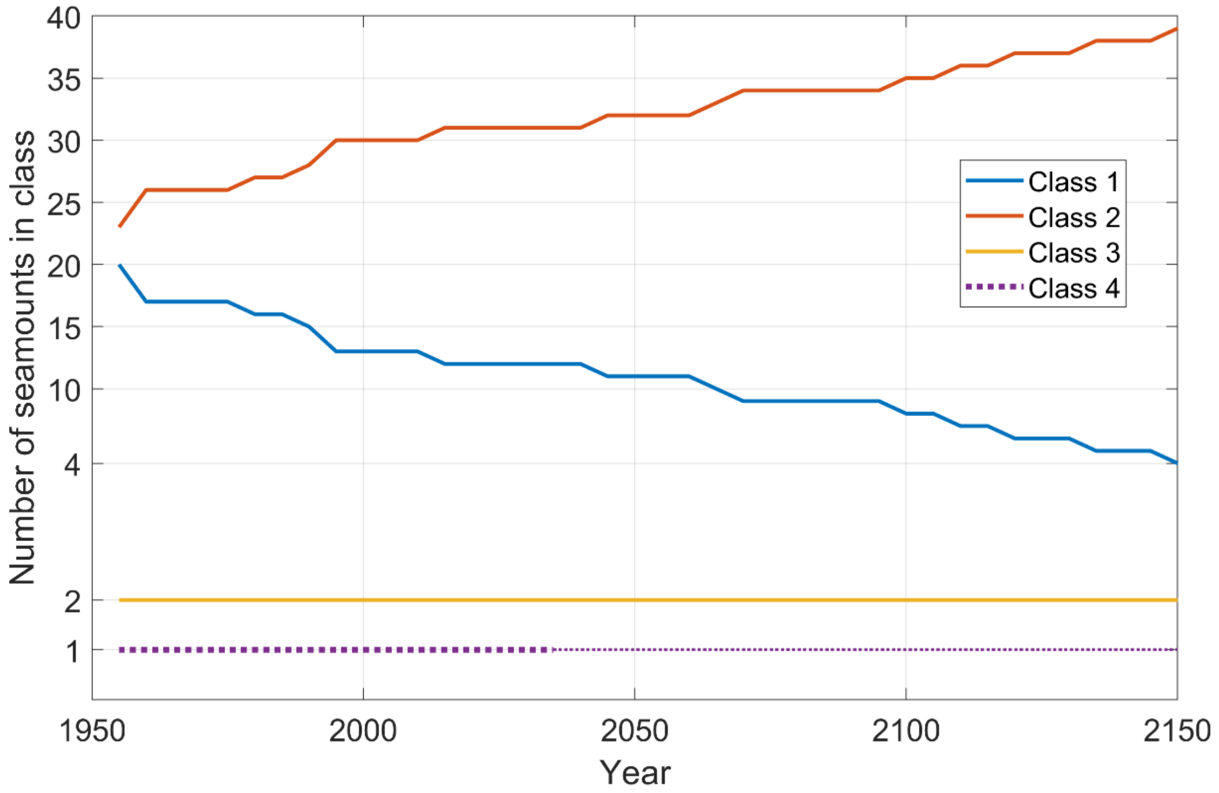

Figure S4: Plot illustrating the potential impact of the observed changes in oxygen on the number of seamounts in the our study region that occupy the different seamount classes identified using Clark et al (2011). Whether the summit is above the calcite saturation horizon is indicated with line thickness (thick, summit is saturated with respect to calcite; thin, summit is under-saturated with respect to all carbonate polymorphs). Post 2019, the assumption is that the trends in the movements of the OMZ boundaries and calcite saturation horizon continue linearly at the rate observed over the last 60 (oxygen) or 30 (carbonate) years.

References:

Clark, M. R., Watling, L., Rowden, A. A., Guinotte, J. M., & Smith, C. R. (2011). A global seamount classification to aid the scientific design of marine protected area networks. *Ocean & Coastal Management*, *54*(1), 19-36. https://doi.org/10.1016/j.ocecoaman.2010.10.006

DFO (2019). Fisheries and Oceans Canada. Biophysical and Ecological Overview of the Offshore Pacific Area of Interest (AOI). DFO Can. Sci. Advis. Sec. Sci. Resp. 2019/011. https://www.dfo-mpo.gc.ca/csas-sccs/Publications/ScR-RS/2019/2019_011-eng.pdf

Dickson, A. G., & Goyet, C. (1994). *Handbook of methods for the analysis of the various parameters of the carbon dioxide system in sea water. Version 2* (No. ORNL/CDIAC-74). Oak Ridge National Lab., TN (United States). https://doi.org/10.2172/10107773

Dickson, A. G., Sabine, C. L., & Christian, J. R. (2007). *Guide to best practices for ocean CO2 measurements*. North Pacific Marine Science Organization. PICES Spec. Publ. 3, 191 pp. Available: https://cdiac.ess-dive.lbl.gov/ftp/oceans/Handbook_2007

Efron, B., & Gong, G. (1983). A leisurely look at the bootstrap, the jackknife, and cross-validation. *The American Statistician*, *37*(1), 36-48. https://doi.org/10.1080/00031305.1983.10483087

Feely, R. A., Sabine, C. L., Lee, K., Berelson, W., Kleypas, J., Fabry, V. J., & Millero, F. J. (2004). Impact of anthropogenic CO2 on the CaCO3 system in the oceans. *Science*, *305*(5682), 362-366. https://doi.org/10.1126/science.1097329

Feely, R. A., Sabine, C. L., Byrne, R. H., Millero, F. J., Dickson, A. G., Wanninkhof, R., ... & Greeley, D. (2012). Decadal changes in the aragonite and calcite saturation state of the Pacific Ocean. *Global Biogeochemical Cycles*, *26*(3). https://doi.org/10.1029/2011GB004157

Fry, C. H., Tyrrell, T., & Achterberg, E. P. (2016). Analysis of longitudinal variations in North Pacific alkalinity to improve predictive algorithms. *Global Biogeochemical Cycles*, *30*(10), 1493-1508. https://doi.org/10.1002/2016GB005398

Ianson, D., Allen, S. E., Harris, S. L., Orians, K. J., Varela, D. E., & Wong, C. S. (2003). The inorganic carbon system in the coastal upwelling region west of Vancouver Island, Canada. *Deep Sea Research Part I: Oceanographic Research Papers*, *50*(8), 1023-1042. https://doi.org/10.1016/S0967-0637(03)00114-6

Lee, K., Tong, L. T., Millero, F. J., Sabine, C. L., Dickson, A. G., Goyet, C., ... & Key, R. M. (2006). Global relationships of total alkalinity with salinity and temperature in surface waters of the world's oceans. *Geophysical research letters*, *33*(19). https://doi.org/10.1029/2006GL027207

Lewis, E. R., & Wallace, D. W. R. (1998). *Program developed for CO2 system calculations* (No. CDIAC: CDIAC-105). ORNL/CDIAC-105, Carbon Dioxide Inf. Anal. Cent., Oak Ridge Natl. Lab., Oak Ridge, Tenn., 38 pp., https://catalog.data.gov/dataset/program-developed-for-co2-system-calculations-program-files-co2sys-calc-dos-v1-05-co2sys-calc-x

Lipsen, M. S., Crawford, D. W., Gower, J., & Harrison, P. J. (2007). Spatial and temporal variability in coccolithophore abundance and production of PIC and POC in the NE subarctic Pacific during El Niño (1998), La Niña (1999) and 2000. *Progress in Oceanography*, *75*(2), 304-325. https://doi.org/10.1016/j.pocean.2007.08.004

Lueker, T. J., Dickson, A. G., & Keeling, C. D. (2000). Ocean pCO2 calculated from dissolved inorganic carbon, alkalinity, and equations for K1 and K2: validation based on laboratory measurements of CO2 in gas and seawater at equilibrium. *Marine chemistry*, 70(1-3), 105-119. https://doi.org/10.1016/S0304-4203(00)00022-0

Millero, F. J., Lee, K., & Roche, M. (1998). Distribution of alkalinity in the surface waters of the major oceans. *Marine Chemistry*, *60*(1-2), 111-130. https://doi.org/10.1016/S0304-4203(97)00084-4

Mucci, A. (1983) The solubility of calcite and aragonite in seawater at various salinities, temperatures and 1 atmosphere total pressure. *Am J Sci* 238: 780–799. https://doi.org/10.2475/ajs.283.7.780

Orr, J. C., Epitalon, J. M., Dickson, A. G., & Gattuso, J. P. (2018). Routine uncertainty propagation for the marine carbon dioxide system. *Marine Chemistry*, *207*, 84-107. https://doi.org/10.1016/j.marchem.2018.10.006

Pitcher, T. J., & Bulman, C. (2007). Raiding the larder: a quantitative evaluation framework and trophic signature for seamount food webs. *Seamounts: Ecology, fisheries and conservation*, 282-295. https://doi.org/10.1002/9780470691953.ch14

Riley, J. P., and M. Tongudai (1967), The major cation/chlorinity ratios in sea water, *Chem. Geol.*, 2, 263–269. https://doi.org/10.1016/0009-2541(67)90026-5

SBE (2012). Seabird Electronics Application Note-64-2: SBE 43 Dissolved Oxygen Sensor Calibration and Data Corrections using Winkler Titrations, 5 pp. https://www.waterproperties.ca/linep/2011-27/documents/Sea-Bird_64-2_Feb2010.pdf

Suzuki, T., Ishii, M., Aoyama, M., Christian, J. R., Enyo, K., Kawano, T., ... & Murata, A. (2013). PACIFICA data synthesis project. *ORNL/CDIAC-159, NDP-092, Carbon Dioxide Information Analysis Center, Oak Ridge National Laboratory, US Department of Energy, Oak Ridge, TN, USA.* Adjustment constants retrieved from: http://pacifica.pices.jp/cgi-bin/PACIFICAadjustment.csv

Uppström, L. R. (1974). The boron/chlorinity ratio of deep-sea water from the Pacific Ocean. *Deep Sea Res.*, 21, 161-162. https://doi.org/10.1016/0011-7471(74)90074-6

Van Heuven, S., Pierrot, D., Rae, J. W. B., Lewis, E., & Wallace, D. W. R. (2011). CO2SYS v 1.1, MATLAB program developed for CO2 system calculations, ORNL/CDIAC-105b. *Carbon Dioxide Information Analysis Center, Oak Ridge National Laboratory, US DoE, Oak Ridge, TN.* https://doi.org/10.3334/CDIAC/otg.CO2SYS_MATLAB_v1.1

Whitney, F. A., Freeland, H. J., & Robert, M. (2007). Persistently declining oxygen levels in the interior waters of the eastern subarctic Pacific. *Progress in Oceanography*, *75*(2), 179-199. https://doi.org/10.1016/j.pocean.2007.08.007
